# Supplementary material for: Systematic review of generative adversarial networks (GANs) in cell microscopy: Trends, practices, and impact on image augmentation
Source: PLoS One. 2025 Jun 24;20(6):e0291217. doi: 10.1371/journal.pone.0291217 (PMC12186945; doi:10.1371/journal.pone.0291217)
Supplement: S1 Table — (PDF) [file pone.0291217.s001.pdf]

S1 Table. Summary of the studies that met the eligibility criteria, full version.

Publication	GAN loss	Generator	Discriminator	Performance metrics	Scores	Model baselines	Data type	Dataset	Training type	Ablation study	Code	Augmentation as main task	D/M	Notes
Wubineh et al. [1] (2025)	Vanilla GAN loss	Regular CNN decoder architecture	Regular CNN encoder architecture	Performance based in downstream task (Classification)	Multi-class scores, refer to publication	No baselines	Optical Microscopy	Private dataset	Unsupervised	No	No	No	No	GAN implementation to boost classification network performance.
Preda et al. [2] (2025)	ACGAN [3]	Regular CNN decoder architecture	SwinT [4]	FID	Multi-class scores, refer to publication	Vanilla GAN, WGAN. **Discriminators:** ResNet [5], ViT [6]	Optical Microscopy	TCGA-CRC-DX [7]	Semi-supervised	Yes	Yes	No	Yes	Ablation study of different discriminators and adversaria losses to find the best architecture to boost classification network performance.
Barrera et al. [8] (2024)	WGAN, Vanilla GAN	Regular CNN decoder architecture, U-Net	Regular CNN encoder architecture, Regular CNN encoder architecture	Performance based in downstream task (Classification)	Multi-class scores. Refer to publication	No baselines	Optical Microscopy	Barrera et al. dataset [9]	Self-supervised	Yes	No	No	Yes	SyntheticCellGAN modification to correct data imbalance for a classification network. The modification includes a new I2I GAN to add stain properties in the image.
Nayar et al. [10] (2024)	Vanilla GAN	Regular CNN decoder architecture	Regular CNN encoder architecture	Performance based in downstream task (Classification)	Acc: 99.1	No baselines	Optical Microscopy	Liquid based-cytology Pap smear dataset [11]	Unsupervised	No	No	No	No	GAN implementation to boost classification network performance.
Khan et al. [12] (2024)	Vanilla GAN	Regular CNN decoder architecture	Regular CNN encoder architecture	Performance based in downstream task (Classification)	Acc > 90	No baselines	Optical Microscopy	PBC dataset [13], LISC dataset [14], and Raabin WBC [15]	Unsupervised	No	No	No	No	GAN implementation to boost classification network performance.
Ngasa et al. [16] (2024)	WGAN-GP	Regular CNN decoder architecture	Regular CNN encoder architecture	FID, IS	FID: 81.9, 11.07 , IS: 9.34, 8.53	WGAN-GP, Improved Denoising Diffusion Probabilistic Models (DDPM) [17]	Optical Microscopy	Private dataset	Unsupervised	Yes	Yes	No	Yes	GAN implementation trained with forwards diffusion samples as inputs. The authors boosted a classification network with the generated images.
Niehues et al. [18] (2024)	Vanilla GAN (with gradient penalty)	StyleGAN2		FID	21.8	Latent diffusion model (LDM) [19], KL-decoder LDM, VQ-decoder LDM	Optical Microscopy	NCT-CRC-HE-100K dataset [20]	Unsupervised	No	Yes	Yes	No	Comparison between three latent diffusion approaches versus StyleGAN2 for histology image generation.
Van Booven et al. [21] (2024)	Vanilla GAN	Regular CNN decoder architecture	Regular CNN encoder architecture	IS	17.2	cGAN, Style-GAN [22]	Optical Microscopy	The Cancer Genomae Atlas (TCGA), Private dataset (available under request)	Unsupervised	No	No	Yes	No	GAN implementation to boost classification network performance.

Publication	GAN loss	Generator	Discriminator	Performance metrics	Scores	Model baselines	Data type	Dataset	Training type	Ablation study	Code	Augmentation as main task	D/M	Notes
Howard et al. [23] (2024)	WGAN-GP, L1 (in W space)	StyleGAN2		Performance based in downstream task (Latent space exploration, reconstruction, feature preservation)		Encoder 4 Editing [24]	Optical Microscopy	TCGA, Clinical Proteomic Tumor Analysis Consortium (CPTAC)	Supervised	No	Yes	Yes	Yes	StyleGAN2 modification. They used a self-supervised encoder as Mapping network. This approach offers several applications.
Khan et al. [25] (2023)	Vanilla GAN	ResNet	ResNet	Performance based in downstream task (Classification)	Multi-class scores. Refer to publication	No baselines	Optical Microscopy	Private dataset, AI-Hub, Liquid based-cytology Pap smear dataset, SIPaKMeD dataset [26]	Unsupervised	Yes	Yes	Yes	Yes	Self-attention GAN implementation to boost cell identification.
Singh et al. [27] (2023)	Vanilla GAN	Regular CNN decoder architecture	Regular CNN encoder architecture	Performance based in downstream task (Classification)	Multi-class scores. Refer to publication	No baselines	Optical Microscopy	BreakHis dataset [28]	Unsupervised	No	No	No	No	GAN used to balance data classes and improve image classification.
Dee et al. [29] (2023)	Conditional WGAN-GP	StyleGAN2-ADA		FID	5.05	No baselines	Optical Microscopy	Tharun and Thompson dataset (available under request) [30], **Niki-TCGA dataset** [31], **The Cancer Genome Atlas (TCGA)**	Supervised	No	Yes	No	No	Boost classification performance of histopathology images.
Gluste et al. [32] (2023)	WGAN-GP, Inspirational GAN (IGAN) [33]	Progressive growing GAN (PGGAN) [34]		Performance based on downstream task (Classification)	AUROC: 0.9659	Improved Denoising Diffusion Probabilistic Models [17]	Optical Microscopy	Private dataset	Unsupervised	No	No	No	No	Two GANs to generate healthy and sick images. They used XAI techniques in the GANs to understand the rejection decision process. Each GAN produce a different latent space (risk and healthy) and they compared the differences in the distributions.
Ghose et al. [35] (2023)	RGAN, L2	PathologyGAN		Performance based on downstream task (Classification)	AUC: 0.82	No baselines	Optical Microscopy	Private dataset (available under request)	Unsupervised	No	Under request	No	No	Study to augment dataset for classification models to detect Ductal carcinoma in situ. They used a latent space representation to select images of interest for the classification network.
Barrera et al. [36] (2022)	WGAN, Vanilla GAN	Regular CNN decoder architecture, U-Net	Regular CNN encoder architecture, Regular CNN encoder architecture	FID, IS, Learned Perceptual Image Patch Similarity (LPIPS)	FID: 12.9–75.7, IS: 9.1–10.6, LPIPS: 0.49–0.53	No baselines	Optical Microscopy	Private dataset	Unsupervised	No	No	Yes	No	Chain GANs implementation. First they use WGAN to produce low-resolution basic mononuclear cells and then a second GAN to do I2I translation to refine the images. The second GAN is in fact 7 GANs trained with a specific cell type.
Kunzmann et al. [37] (2022)	Conditional Vanilla GAN	Vanilla GAN		Qualitative assessment		Latent diffusion model [19]	Optical Microscopy	**Asthma Equidae dataset** [38]	Supervised	No	No	Yes	No	Compares the performance of a diffusion model and a GAN to see the potential of synthetic new data.
Dolezal et al. [39] (2022)	Conditional WGAN	StyleGAN2		FID, qualitative assessment	3.67, 4.46, 5.19	No baselines	Optical Microscopy	**TCGA, Clinical Proteomic Tumor Analysis Consortium (CPTAC)**	Supervised	No	Yes	No	No	Uses generated data to then do XAI on classifier of cell lung cancer cells.

Publication	GAN loss	Generator	Discriminator	Performance metrics	Scores	Model baselines	Data type	Dataset	Training type	Ablation study	Code	Augmentation as main task	D/M	Notes
Rando et al. [40] (2022)	Vanilla GAN	Regular CNN decoder architecture	Regular CNN encoder architecture	FID, IS	FID: 80.58, IS: 1.62	No baselines	Optical Microscopy	Private dataset	Unsupervised	No	No	No	No	DCGAN trained to augment dataset and boost performance classification method for cervical cancer.
Pandya et al. [41] (2022)	Vanilla GAN	Regular CNN decoder architecture	Regular CNN encoder architecture	No metrics		No baselines	Optical Microscopy	Leukemia dataset	Unsupervised	No	No	Yes	No	Augment white blood cells dataset.
Liu et al. [42] (2021)	WGAN-GP	StyleGAN		IS	1.78	DC-GAN [43], Pro-GAN [44]	Optical Microscopy	**BCCD** [45]	Unsupervised	Yes	No	Yes	No	Mix between GAN and VAE. Uses the encoder of VAE to generate the Z latent space using the images as input.
Yu et al. [46] (2021)	Vanilla GAN	Regular CNN decoder architecture	Regular CNN encoder architecture	Performance based on downstream task (Classification)	Acc: 0.938, Precision: 0.478, AUC: 0.984	No baselines	Optical Microscopy	Private dataset	Unsupervised	No	No	No	No	Uses GANs to augment the dataset to fix the imbalance in abnormal cells for cervical cancer classification.
Zhao et al. [47] (2021)	WGAN	Regular CNN decoder architecture	Regular CNN encoder architecture	FID	257.64, 296.6, 178.11, 219.87, 164.51	No GAN baselines	Optical Microscopy	**Zheng et al.** [48], Private dataset	Unsupervised	No	No	No	No	Image augmentation with WGAN to boost a classification network.
Mirzazadeh et al. [49] (2021)	WGAN-GP	PGGAN		Mattthews correlation coefficient (MCC) in the classifier	0.79	No baselines	Optical Microscopy	**DNA-based transplant rejection** [50], **Children's hospital of Atlanta** [51] [52] [53] [54]	Unsupervised	No	No	No	No	Uses GAN to boost the performance of a rejection classification network.
Quiros et al. [55] (2020)	RGAN, L2	PathologyGAN		Qualitative assessment		No baselines	Optical Microscopy	**Netherlands Cancer Institute** [56], **Vancouver General Hospital databases** [57]	Unsupervised	No	No	Yes	Yes	The GAN architecture includes a backward encoder to get a latent space representation of the generated image to extract image features. The goal is also to be able to map real images into the latent space to extract features.
Murali et al. [58] (2020)	Vanilla GAN	Regular CNN decoder architecture	Regular CNN encoder architecture	FID, qualitative assessment	Around 514	No baselines	Optical Microscopy	Private dataset	Unsupervised	No	No	Yes	No	Uses GANs to get synthetic visualization of samples with different stains. Pipeline of DCGAN + ESRGAN [59] + CycleGAN.
Teramoto et al. [60] (2020)	WGAN	PGGAN		Accuracy, Sensitivity, Specificity (downstream task)	Acc: 0.85, Sensitivity: 0.854, Specificity: 0.853	DC-GAN	Optical Microscopy	Private dataset	Unsupervised	No	No	No	No	Does image augmentation to boost the performance of a classifier network.
Almezhghwi et al. [61] (2020)	Vanilla GAN	Vanilla GAN		Performance based on downstream task (Classification)	Acc: 0.988	No baselines	Optical Microscopy	**LISC dataset** [14]	Unsupervised	No	No	No	No	Uses GANs to augment white cell types datasets (imbalance) and they also explore if using pretrained networks in other datasets is beneficial (in classification).
Chen et al. [62] (2020)	WGAN-GP, L2	ResNet [5]	Regular CNN encoder architecture	FID	13.004	Vanilla GAN	Optical Microscopy	**Pap-smear dataset** [63]	Supervised	Yes	No	Yes	Yes	Makes a conditional data (labels) and increases the amount of data available.
Wang et al. [64] (2019)	WGAN	WGAN		Qualitative assessment		No baselines	Optical Microscopy	Private dataset	Unsupervised	No	No	Yes	No	Image augmentation to boost the performance of a classification network (AlexNet-styled).
Bo et al. [65] (2018)	WGAN-GP, Mutual information (MI)	ResNet	ResNet	Performance based on downstream tasks (Segmentation, classification and clustering)		No baselines	Optical Microscopy	**BM dataset** [66], Private dataset	Unsupervised	No	Yes	No	Yes	Adds an auxiliary network to the GAN to encourage mutual information between part of the generator input and the images.

Publication	GAN loss	Generator	Discriminator	Performance metrics	Scores	Model baselines	Data type	Dataset	Training type	Ablation study	Code	Augmentation as main task	D/M	Notes
Tang et al. [67] (2024)	Vanilla GAN (with gradient penalty)	StyleGAN2		FID	3.04 (mask), 3.67 (image)	DC-GAN, PG-GAN	Fluorescence microscopy	smFISH dataset [68–70]	Unsupervised	No	Yes	Yes	No	Two sequential GANs for segmentation network augmentation. First GAN produce segmentation masks and the second produce the final images.
Mascolini et al. [71] (2022)	WGAN, Jacobian Regularization, L1, R1 regularization [72]	StyleGAN2		Accuracy	>0.91	WGAN, Pretrained CNN (ImageNet)	Fluorescence microscopy	**RxRx19a Sars-CoV-2 image collection** [73], **RxRx1 dataset** [74]	Self-supervised	No	No	Yes	Yes	WGAN and SVM combination.
Eschweiler et al. [75] (2021)	cGAN, L1	3D U-Net [76]	PatchGAN	normalized root mean squared error (NRMSE), structural similarity index measure (SSIM), Zero mean normalized cross-correlation (ZNCC)	NRMSE: 0.125, SSIM: 0.641, ZNCC: 0.742	No baselines	Fluorescence microscopy	**Willis et al.** [77], **Faure et al.** [78]	Unsupervised	Yes	Yes	Yes	Yes	3D fluorescence microscopy. Although a I2I task, they synthesized the GAN input images themselves.
Reich et al. [79] (2021)	Vanilla GAN, R1 regularization, Path length [80]	Two parallel convolutional paths (StyleGAN generator) for each domain style	U-Net with adaptive discriminator augmentation	IS, FID, Fréchet Video distance	IS: 1.864, 2.437; FID: 33.37, 207.84; FVD: 4.46, 30.16	StyleGAN2, StyleGAN2 3D	Time-lapse fluorescence microscopy	Private dataset	Unsupervised	Yes	Yes	Yes	Yes	Based on StyleGAN2 and star-shaped GAN. Pretends to produce time series of images.
Tasnadi et al. [81] (2023)	Vanilla GAN (with gradient penalty)	StyleGAN2		FID	108.79, 61.99	No baselines	Immunofluorescence microscopy	Salivary Gland Tumor and Fallopian datasets [82]	Unsupervised	No	Yes	No	No	Two sequential GANs for segmentation network augmentation. First GAN produce segmentation masks and the second produce the final images.
Anaam et al. [83] (2023)	WGAN-GP, MI	ResNet	ResNet	FID	22.3	DC-GAN, WGAN, WGAN-GP, Info-GAN [84]	Immunofluorescence microscopy	I3A dataset [85]	Unsupervised	No	Yes	No	No	Info-WGANGP implementation for classification network augmentation.
Anaam et al. [86] (2023)	WGAN-GP, MI	ResNet	ResNet	FID	10.64	No baselines	Immunofluorescence microscopy	**I3A dataset** [85]	Supervised	No	No	No	No	Implementation of infoGAN with WGAN-GP loss. Boost classification performance.
Anaam et al. [87] (2021)	WGAN-GP, MI	ResNet	Two output layers CNN	FID, Classifier two-sample test (C2ST)	Multi-class scores. Refer to publication	DC-GAN, WGAN, WGAN-GP	Immunofluorescence microscopy	**I3A dataset**	Unsupervised	Yes	No	Yes	Yes	Tests different GAN architectures to see which improves the performance of a classifier network.
Dimitrakopoulos et al. [88] (2020)	Vanilla GAN, Markov Random Field-based loss	Regular CNN decoder architecture	Regular CNN encoder architecture	FID, IoU in auxiliary segmentation models trained with real and generated data	IoU: >0.55, FID: 88	Vanilla GAN, No GAN-based augmentation	Fluorescence microscopy, Optical microscopy	**BBBC038v1 dataset** [89]	Unsupervised	No	No	Yes	Yes	A single GAN with sibling branches to produce both microscopy image and corresponding segmentation mask.
Verma et al. [90] (2020)	Vanilla GAN	Regular CNN decoder architecture	Regular CNN encoder architecture	Performance based on downstream task (Classification)	Acc: 0.906, Micro F1: 0.894, Macro F1: 0.834	No baselines	Fluorescence microscopy	**Human Protein Atlas Image Classification Kaggle competition** [91]	Unsupervised	No	No	Yes	No	Tests how well can GANs improve the performance of CNN classifier (difficulty in protein image acquisition).
Hussain et al. [92] (2020)	Vanilla GAN	Regular CNN decoder architecture	Regular CNN encoder architecture	Classification Accuracy, Kolmogorov–Smirnov (KS) distance of the first principal component of features extracted by additional feature extraction network	>0.97	Vanilla-GAN, ProGAN	Widefield fluorescence microscopy	Private dataset	Unsupervised	No	No	Yes	No	Compares three architectures to find the one that best produces synthetic data (Vanilla, DCGAN and ProGAN). Tests the model to generate cell and bacteria images. The classification module identifies drug-induced anomalous effects.

**Publication**	**GAN loss**	**Generator**	**Discriminator**	**Performance metrics**	**Scores**	**Model baselines**	**Data type**	**Dataset**	**Training type**	**Ablation study**	**Code**	**Augmentation as main task**	**D/M**	**Notes**
Kastaniotis et al. [93] (2018)	Vanilla GAN, L2	Regular CNN decoder architecture	Regular CNN encoder with attention map generation	Qualitative assessment		No baselines	Fluorescence microscopy	**Hep-2 Cells Classification contest** [94]	Unsupervised	No	Yes	Yes	Yes	Uses transfer knowledge with attention maps from classification trained network to guide the task of the discriminator.
Osokin et al. [95] (2017)	WGAN-GP	Star-shaped generator	Regular CNN encoder architecture	C2ST	1.6	Vanilla GAN, WGAN	Fluorescence microscopy	**LIN dataset** [96]	Unsupervised	Yes	Yes	Yes	Yes	Makes a multichannel GAN to generate channels by separate.
Devan et al. [97] (2021)	WGAN-GP	sinGAN [98]		Performance based on downstream task (Object detection)	AP: 0.7648	No baselines	Transmission electron microscopy	Private dataset (available under request)	Unsupervised	No	Yes	No	No	Uses of only 10 image samples during GAN training.
Han et al. [99] (2018)	Vanilla GAN	Regular CNN decoder architecture	Multi-scale PatchGAN	mean IoU, average cell size, average mitochondria size and roundness, average number of mitochondrias per cell, realisticness (SVM real, fake classifier)	Metrics related to the synthesized microscopy image, not the label image (image augmentation part))	Zhao et al. Pipeline [100], Non-parametric baseline, DC-GAN	Transmission Electron Microscopy	**VNC dataset** [101]	Supervised	Yes	Yes	Yes	Yes	It first takes input noise to produce the synthetic labels with regular GAN, and later use the synthesized label to generate its cell image with conditional GAN.
Rubin et al. [102] (2018)	Vanilla GAN	Regular CNN decoder architecture	Regular CNN encoder architecture	Sensitivity, Specificity, AUC (for the classification task)	Sensitivity: 0.98, 0.93; Specificity: 0.99, 0.93; AUC: 0.995, 0.947	No GAN baselines (metrics based on classification only)	interferometric phase microscopy	Private dataset	Unsupervised	No	No	No	No	Uses transfer learning to augment small datasets with modalities. Image augmentation is used to boost classification network performance.

References

[1] Wubineh BZ, Jeleń Ł, Rusiecki A. DCGAN-based Cytology Image Augmentation for Cervical Cancer Cell Classification Using Transfer Learning. Procedia Computer Science. 2025;256:1003–1011. doi:10.1016/j.procs.2025.02.206.

[2] Preda AA, Tăiatu IM, Cercel DC. Scaling Federated Learning Solutions with Kubernetes for Synthesizing Histopathology Images; 2025.

[3] Odena A, Olah C, Shlens J. Conditional Image Synthesis with Auxiliary Classifier GANs. In: Proceedings of the 34th International Conference on Machine Learning. vol. 70 of Proceedings of Machine Learning Research. PMLR; 2017. p. 2642–2651.

[4] Liu Z, Lin Y, Cao Y, Hu H, Wei Y, Zhang Z, et al. Swin Transformer: Hierarchical Vision Transformer Using Shifted Windows. In: Proceedings of the IEEE/CVF International Conference on Computer Vision (ICCV); 2021. p. 10012–10022.

[5] He K, Zhang X, Ren S, Sun J. Deep Residual Learning for Image Recognition. In: 2016 IEEE Conference on Computer Vision and Pattern Recognition (CVPR); 2016. p. 770–778.

[6] Dosovitskiy A, Beyer L, Kolesnikov A, Weissenborn D, Zhai X, Unterthiner T, et al. An Image Is Worth 16x16 Words: Transformers for Image Recognition at Scale. In: International Conference on Learning Representations; 2021.

[7] Kather JN. Histological Image Tiles for TCGA-CRC-DX, Color-Normalized, Sorted by MSI Status, Train/Test Split; 2020.

[8] Barrera K, Rodellar J, Alférez S, Merino A. A Deep Learning Approach for Automatic Recognition of Abnormalities in the Cytoplasm of Neutrophils. Computers in Biology and Medicine. 2024;178:108691. doi:10.1016/j.compbiomed.2024.108691.

[9] Barrera Llanga KI, Rodellar J, Alferez S, Merino A. A Deep Learning Approach for Automatic Recognition of Abnormalities in the Cytoplasm of Neutrophils - Dataset; 2024.

[10] Nayar S, Priya D, J VP. Deep Learning Approach for Automated Data Augmentation and Multi-Class Classification of Pap Smear Images. Procedia Computer Science. 2024;235:2205–2214. doi:10.1016/j.procs.2024.04.209.

[11] Hussain E, Mahanta LB, Borah H, Das CR. Liquid Based-Cytology Pap Smear Dataset for Automated Multi-Class Diagnosis of Pre-Cancerous and Cervical Cancer Lesions. Data in Brief. 2020;30:105589. doi:10.1016/j.dib.2020.105589.

[12] Khan S, Sajjad M, Abbas N, Escorcia-Gutierrez J, Gamarra M, Muhammad K. Efficient Leukocytes Detection and Classification in Microscopic Blood Images Using Convolutional Neural Network Coupled with a Dual Attention Network. Computers in Biology and Medicine. 2024;174:108146. doi:10.1016/j.compbiomed.2024.108146.

[13] Acevedo A, Merino A, Alférez S, Molina Á, Boldú L, Rodellar J. A Dataset of Microscopic Peripheral Blood Cell Images for Development of Automatic Recognition Systems. Data in Brief. 2020;30:105474. doi:10.1016/j.dib.2020.105474.

[14] Rezatofighi SH, Soltanian-Zadeh H. Automatic Recognition of Five Types of White Blood Cells in Peripheral Blood. Computerized Medical Imaging and Graphics. 2011;35(4):333–343. doi:10.1016/j.compmedimag.2011.01.003.

[15] Kouzehkanan ZM, Saghari S, Tavakoli S, Rostami P, Abaszadeh M, Mirzadeh F, et al. A Large Dataset of White Blood Cells Containing Cell Locations and Types, along with Segmented Nuclei and Cytoplasm. Scientific Reports. 2022;12(1):1123. doi:10.1038/s41598-021-04426-x.

[16] Ngasa EE, Jang MA, Tarimo SA, Woo J, Shin HB. Diffusion-Based Wasserstein Generative Adversarial Network for Blood Cell Image Augmentation. Engineering Applications of Artificial Intelligence. 2024;133:108221. doi:10.1016/j.engappai.2024.108221.

[17] Nichol AQ, Dhariwal P. Improved Denoising Diffusion Probabilistic Models. In: Proceedings of the 38th International Conference on Machine Learning. PMLR; 2021. p. 8162–8171.

[18] Niehues JM, Müller-Franzes G, Schirris Y, Wagner SJ, Jendrusch M, Kloor M, et al. Using Histopathology Latent Diffusion Models as Privacy-Preserving Dataset Augmenters Improves Downstream Classification Performance. Computers in Biology and Medicine. 2024;175:108410. doi:10.1016/j.compbiomed.2024.108410.

[19] Rombach R, Blattmann A, Lorenz D, Esser P, Ommer B. High-Resolution Image Synthesis with Latent Diffusion Models. In: Proceedings of the IEEE/CVF Conference on Computer Vision and Pattern Recognition. IEEE Computer Society; 2022. p. 10684–10695.

[20] Kather JN, Halama N, Marx A. 100,000 Histological Images of Human Colorectal Cancer and Healthy Tissue;.

[21] Van Booven DJ, Chen CB, Kryvenko O, Punnen S, Sandoval V, Malpani S, et al. Synthetic Histology Images for Training AI Models: A Novel Approach to Improve Prostate Cancer Diagnosis. bioRxiv : the preprint server for biology. 2024;doi:10.1101/2024.01.25.577225.

[22] Karras T, Laine S, Aila T. A Style-Based Generator Architecture for Generative Adversarial Networks. In: 2019 IEEE/CVF Conference on Computer Vision and Pattern Recognition (CVPR); 2019. p. 4401–4410.

[23] Howard FM, Hieromnimon HM, Ramesh S, Dolezal J, Kochanny S, Zhang Q, et al. Generative Adversarial Networks Accurately Reconstruct Pan-Cancer Histology from Pathologic, Genomic, and Radiographic Latent Features. Science Advances. 2024;10(46):eadq0856. doi:10.1126/sciadv.adq0856.

[24] Tov O, Alaluf Y, Nitzan Y, Patashnik O, Cohen-Or D. Designing an Encoder for Stylegan Image Manipulation. ACM Transactions on Graphics (TOG). 2021;40(4):1–14.

[25] Khan A, Han S, Ilyas N, Lee YM, Lee B. CervixFormer: A Multi-scale Swin Transformer-Based Cervical Pap-Smear WSI Classification Framework. Computer Methods and Programs in Biomedicine. 2023;240:107718. doi:10.1016/j.cmpb.2023.107718.

[26] Plissiti ME, Dimitrakopoulos P, Sfikas G, Nikou C, Krikoni O, Charchanti A. Sipakmed: A New Dataset for Feature and Image Based Classification of Normal and Pathological Cervical Cells in Pap Smear Images. In: 2018 25th IEEE International Conference on Image Processing (ICIP); 2018. p. 3144–3148.

[27] Singh UP, Sahu M. A Hybrid Approach for Improving the Classification Performance of Imbalanced Breast Cancer Data. In: 2023 International Conference on Communication, Circuits, and Systems (IC3S); 2023. p. 1–6.

[28] Spanhol FA, Oliveira LS, Petitjean C, Heutte L. A Dataset for Breast Cancer Histopathological Image Classification. IEEE Transactions on Biomedical Engineering. 2016;63(7):1455–1462. doi:10.1109/TBME.2015.2496264.

[29] Dee W, Ibrahim RA, Marouli E. Histopathological Domain Adaptation with Generative Adversarial Networks Bridging the Domain Gap Between Thyroid Cancer Histopathology Datasets; 2023.

[30] Böhland M, Tharun L, Scherr T, Mikut R, Hagenmeyer V, Thompson LDR, et al. Machine Learning Methods for Automated Classification of Tumors with Papillary Thyroid Carcinoma-like Nuclei: A Quantitative Analysis. PLOS ONE. 2021;16(9):1–21. doi:10.1371/journal.pone.0257635.

[31] Nikiforov YE, Seethala RR, Tallini G, Baloch ZW, Basolo F, Thompson LDR, et al. Nomenclature Revision for Encapsulated Follicular Variant of Papillary Thyroid Carcinoma: A Paradigm Shift to Reduce Overtreatment of Indolent Tumors. JAMA Oncology. 2016;2(8):1023–1029. doi:10.1001/jamaoncol.2016.0386.

[32] Giuste FO, Sequeira R, Keerthipati V, Lais P, Mirzazadeh A, Mohseni A, et al. Explainable Synthetic Image Generation to Improve Risk Assessment of Rare Pediatric Heart Transplant Rejection. Journal of Biomedical Informatics. 2023;139:104303. doi:10.1016/j.jbi.2023.104303.

[33] Rozière B, Riviere M, Teytaud O, Rapin J, LeCun Y, Couprie C. Inspirational Adversarial Image Generation. IEEE Transactions on Image Processing. 2021;30:4036–4045. doi:10.1109/TIP.2021.3065845.

[34] Karras T, Aila T, Laine S, Lehtinen J. Progressive Growing of GANs for Improved Quality, Stability, and Variation. In: International Conference on Learning Representations; 2018.

[35] Ghose S, Cho S, Ginty F, McDonough E, Davis C, Zhang Z, et al. Predicting Breast Cancer Events in Ductal Carcinoma In Situ (DCIS) Using Generative Adversarial Network Augmented Deep Learning Model. Cancers. 2023;15(7):1922. doi:10.3390/cancers15071922.

[36] Barrera K, Merino A, Molina A, Rodellar J. Automatic Generation of Artificial Images of Leukocytes and Leukemic Cells Using Generative Adversarial Networks (Syntheticcellgan). Computer Methods and Programs in Biomedicine. 2023;229:107314. doi:10.1016/j.cmpb.2022.107314.

[37] Kunzmann S, Öttl M, Madhu P, Denzinger F, Maier A. An Unobtrusive Quality Supervision Approach for Medical Image Annotation; 2022.

[38] Marzahl C, Bertram CA, Wilm F, Voigt J, Barton AK, Klopfleisch R, et al. Cell Detection for Asthma on Partially Annotated Whole Slide Images. In: Palm C, Deserno TM, Handels H, Maier A, Maier-Hein K, Tolxdorff T, editors. Bildverarbeitung für die Medizin 2021. Wiesbaden: Springer Fachmedien; 2021. p. 147–152.

[39] Dolezal JM, Wolk R, Hieromnimon HM, Howard FM, Srisuwananukorn A, Karpeyev D, et al. Deep Learning Generates Synthetic Cancer Histology for Explainability and Education. npj Precision Oncology. 2023;7(1):1–13. doi:10.1038/s41698-023-00399-4.

[40] Rando, Setiawan NA, Permanasari AE, Rulaningtyas R, Suksmono AB, Sitanggang IS. DCGAN-based Medical Image Augmentation to Improve ELM Classification Performance. In: 2022 IEEE International Conference on Communication, Networks and Satellite (COMNETSAT); 2022. p. 206–211.

[41] Pandya D, Patel T, kumar Singh D. White Blood Cell Image Generation Using Deep Convolutional Generative Adversarial Network. In: 2022 International Conference on Augmented Intelligence and Sustainable Systems (ICAISS); 2022. p. 129–134.

[42] Liu K, Shuai R, Ma L, ZeXu. Cells Image Generation Method Based on VAE-SGAN. Procedia Computer Science. 2021;183:589–595. doi:10.1016/j.procs.2021.02.101.

[43] Radford A, Metz L, Chintala S. Unsupervised Representation Learning with Deep Convolutional Generative Adversarial Networks. In: Bengio Y, LeCun Y, editors. 4th International Conference on Learning Representations, ICLR 2016, San Juan, Puerto Rico, May 2-4, 2016, Conference Track Proceedings. arXiv; 2016.

[44] Gao H, Pei J, Huang H. ProGAN: Network Embedding via Proximity Generative Adversarial Network. In: Proceedings of the 25th ACM SIGKDD International Conference on Knowledge Discovery & Data Mining. KDD '19. New York, NY, USA: Association for Computing Machinery; 2019. p. 1308–1316.

[45] shenggan. BCCD Dataset; 2017.

[46] Yu S, Zhang S, Wang B, Dun H, Xu L, Huang X, et al. Generative Adversarial Network Based Data Augmentation to Improve Cervical Cell Classification Model. Mathematical Biosciences and Engineering. 2021;18(2):1740–1752. doi:10.3934/mbe.2021090.

[47] Zhao M, Jin L, Teng S, Li Z. Attention Residual Network for White Blood Cell Classification with WGAN Data Augmentation. In: 2021 11th International Conference on Information Technology in Medicine and Education (ITME); 2021. p. 336–340.

[48] Zheng X, Wang Y, Wang G. White Blood Cell Segmentation Using Expectation-Maximization and Automatic Support Vector Machine Learning. Data Acquisition and Processing. 2013;28(5):614–619.

[49] Mirzazadeh A, Mohseni A, Ibrahim S, Giuste FO, Zhu Y, Shehata BM, et al. Improving Heart Transplant Rejection Classification Training Using Progressive Generative Adversarial Networks. In: 2021 IEEE EMBS International Conference on Biomedical and Health Informatics (BHI); 2021. p. 1–4.

[50] Zangwill SD, Kindel SJ, Schroder JN, Bichell DP, Deshpande SR, Wigger MA, et al. Increase in Total Cell-Free DNA Correlates with Death in Adult and Pediatric Heart Transplant Recipients: DNA Based Transplant Rejection Test (DTRT)-A Prospective Blinded Multicenter NIH/NHLBI Funded Clinical Study. The Journal of Heart and Lung Transplantation. 2019;38(4, Supplement):S50–S51. doi:10.1016/j.healun.2019.01.110.

[51] Dooley AE, Tong L, Deshpande SR, Wang MD. Prediction of Heart Transplant Rejection Using Histopathological Whole-Slide Imaging. In: 2018 IEEE EMBS International Conference on Biomedical & Health Informatics (BHI); 2018. p. 251–254.

[52] Giuste F, Venkatesan M, Zhao C, Tong L, Zhu Y, Deshpande SR, et al. Automated Classification of Acute Rejection from Endomyocardial Biopsies. In: Proceedings of the 11th ACM International Conference on Bioinformatics, Computational Biology and Health Informatics. BCB '20. New York, NY, USA: Association for Computing Machinery; 2020. p. 1–9.

[53] Tong L, Hoffman R, Deshpande SR, Wang MD. Predicting Heart Rejection Using Histopathological Whole-Slide Imaging and Deep Neural Network with Dropout. In: 2017 IEEE EMBS International Conference on Biomedical and Health Informatics (BHI); 2017. p. 1–4.

[54] Zhu Y, Tong L, Deshpande SR, Wang MD. Improved Prediction on Heart Transplant Rejection Using Convolutional Autoencoder and Multiple Instance Learning on Whole-Slide Imaging. In: 2019 IEEE EMBS International Conference on Biomedical and Health Informatics (BHI); 2019. p. 1–4.

[55] Quiros AC, Murray-Smith R, Yuan K. Learning a Low Dimensional Manifold of Real Cancer Tissue with PathologyGAN. In: CoRR. vol. abs/2004.06517; 2020.

[56] Liu R, Wang X, Chen GY, Dalerba P, Gurney A, Hoey T, et al. The Prognostic Role of a Gene Signature from Tumorigenic Breast-Cancer Cells. New England Journal of Medicine. 2007;356(3):217–226. doi:10.1056/NEJMoa063994.

[57] Hitchcock CL. The Future of Telepathology for the Developing World. Archives of Pathology & Laboratory Medicine. 2011;135(2):211–214. doi:10.5858/135.2.211.

[58] Murali LK, Lutnick B, Ginley B, Tomaszewski JE, Sarder P. Generative Modeling for Renal Microanatomy. In: Tomaszewski JE, Ward AD, editors. Proceedings of SPIE–the International Society for Optical Engineering. vol. 11320. SPIE / International Society for Optics and Photonics; 2020. p. 113200F.

[59] Wang X, Yu K, Wu S, Gu J, Liu Y, Dong C, et al. ESRGAN: Enhanced Super-Resolution Generative Adversarial Networks. In: Computer Vision – ECCV 2018 Workshops. Cham: Springer International Publishing; 2019. p. 63–79.

[60] Teramoto A, Tsukamoto T, Yamada A, Kiriyama Y, Imaizumi K, Saito K, et al. Deep Learning Approach to Classification of Lung Cytological Images: Two-step Training Using Actual and Synthesized Images by Progressive Growing of Generative Adversarial Networks. PLOS ONE. 2020;15(3):1–12. doi:10.1371/journal.pone.0229951.

[61] Almezhghwi K, Serte S. Improved Classification of White Blood Cells with the Generative Adversarial Network and Deep Convolutional Neural Network. Computational Intelligence and Neuroscience. 2020;2020:6490479. doi:10.1155/2020/6490479.

[62] Chen S, Gao D, Wang L, Zhang Y. Cervical Cancer Single Cell Image Data Augmentation Using Residual Condition Generative Adversarial Networks. In: 2020 3rd International Conference on Artificial Intelligence and Big Data (ICAIBD); 2020. p. 237–241.

[63] Jantzen J, Norup J, Dounias G, Bjerregaard B. Pap-Smear Benchmark Data for Pattern Classification. In: Proc. NiSIS 2005. NiSIS; 2005. p. 1–9.

[64] Wang D, Lu Z, Bao Z. Augmenting C. Elegans Microscopic Dataset for Accelerated Pattern Recognition; 2019.

[65] Hu B, Tang Y, Chang EIC, Fan Y, Lai M, Xu Y. Unsupervised Learning for Cell-Level Visual Representation in Histopathology Images With Generative Adversarial Networks. IEEE Journal of Biomedical and Health Informatics. 2019;23(3):1316–1328. doi:10.1109/JBHI.2018.2852639.

[66] Kainz P, Urschler M, Schulter S, Wohlhart P, Lepetit V. You Should Use Regression to Detect Cells. In: Navab N, Hornegger J, Wells WM, Frangi AF, editors. Medical Image Computing and Computer-Assisted Intervention – MICCAI 2015. Cham: Springer International Publishing; 2015. p. 276–283.

[67] Tang J, Du W, Shu Z, Cao Z. A Generative Benchmark for Evaluating the Performance of Fluorescent Cell Image Segmentation. Synthetic and Systems Biotechnology. 2024;9(4):627–637. doi:10.1016/j.synbio.2024.05.005.

[68] Zuckerman B, Ulitsky I. smFISH Data for Mol. Cell: NORAD and NXF1 Protein Co-Staining; 2020.

[69] Zuckerman B, Ulitsky I. smFISH Data for Mol. Cell: NORAD, MALAT1 and Oligo-dT Probes; siALY+UAP56; 2020.

[70] Zuckerman B, Ulitsky I. smFISH Data for Mol. Cell: NORAD, MALAT1 and Oligo-dT Probes; siNXF1; 2020.

[71] Mascolini A, Cardamone D, Ponzio F, Di Cataldo S, Ficarra E. Exploiting Generative Self-Supervised Learning for the Assessment of Biological Images with Lack of Annotations. BMC Bioinformatics. 2022;23(1):295. doi:10.1186/s12859-022-04845-1.

[72] Mescheder L, Geiger A, Nowozin S. Which Training Methods for GANs Do Actually Converge? In: Proceedings of the 35th International Conference on Machine Learning. PMLR; 2018. p. 3481–3490.

[73] RxRx19a Dataset;. https://www.rxrx.ai/rxrx19a.

[74] RxRx1 Dataset;. https://www.rxrx.ai/rxrx1.

[75] Eschweiler D, Rethwisch M, Jarchow M, Koppers S, Stegmaier J. 3D Fluorescence Microscopy Data Synthesis for Segmentation and Benchmarking. PLOS ONE. 2021;16(12):1–21. doi:10.1371/journal.pone.0260509.

[76] Çiçek Ö, Abdulkadir A, Lienkamp SS, Brox T, Ronneberger O. 3D U-Net: Learning Dense Volumetric Segmentation from Sparse Annotation. In: Medical Image Computing and Computer-Assisted Intervention – MICCAI 2016. Cham: Springer International Publishing; 2016. p. 424–432.

[77] Willis L, Refahi Y, Wightman R, Landrein B, Teles J, Huang KC, et al. Cell Size and Growth Regulation in the Arabidopsis Thaliana Apical Stem Cell Niche. Proceedings of the National Academy of Sciences. 2016;113(51):E8238–E8246. doi:10.1073/pnas.1616768113.

[78] Faure E, Savy T, Rizzi B, Melani C, Stašová O, Fabrèges D, et al. A Workflow to Process 3D+time Microscopy Images of Developing Organisms and Reconstruct Their Cell Lineage. Nature Communications. 2016;7(1):8674. doi:10.1038/ncomms9674.

[79] Reich C, Prangemeier T, Wildner C, Koeppl H. Multi-StyleGAN: Towards Image-Based Simulation of Time-Lapse Live-Cell Microscopy. In: de Bruijne M, Cattin PC, Cotin S, Padoy N, Speidel S, Zheng Y, et al., editors. Medical Image Computing and Computer Assisted Intervention – MICCAI 2021. Cham: Springer International Publishing; 2021. p. 476–486.

[80] Schonfeld E, Schiele B, Khoreva A. A U-Net Based Discriminator for Generative Adversarial Networks. In: Proceedings of the IEEE/CVF Conference on Computer Vision and Pattern Recognition. IEEE Computer Society; 2020. p. 8207–8216.

[81] Tasnadi E, Sliz-Nagy A, Horvath P. Structure Preserving Adversarial Generation of Labeled Training Samples for Single-Cell Segmentation. Cell Reports Methods. 2023;3(9). doi:10.1016/j.crmeth.2023.100592.

[82] Tasnadi E, Sliz-Nagy A, Horvath P. Structure Preserving Adversarial Generation of Labeled Training Samples for Single Cell Segmentation; 2023.

[83] Anaam A, Al-antari MA, Gofuku A. A Deep Learning Self-Attention Cross Residual Network with Info-WGANGP for Mitotic Cell Identification in HEp-2 Medical Microscopic Images. Biomedical Signal Processing and Control. 2023;86:105191. doi:10.1016/j.bspc.2023.105191.

[84] Chen X, Duan Y, Houthooft R, Schulman J, Sutskever I, Abbeel P. InfoGAN: Interpretable Representation Learning by Information Maximizing Generative Adversarial Nets. In: Lee D, Sugiyama M, Luxburg U, Guyon I, Garnett R, editors. Advances in Neural Information Processing Systems. vol. 29. Curran Associates, Inc.; 2016.

[85] Hobson P, Percannella G, Vento M, Wiliem A. Competition on Cells Classification by Fluorescent Image Analysis. In: 2013 IEEE International Conference on Image Processing; 2013.

[86] Anaam A, Al-Antari MA, Gofuku A. A Hybrid Ensemble Learning with Generative Adversarial Networks for HEp-2 Cell Image Classification. In: 2022 IEEE-EMBS Conference on Biomedical Engineering and Sciences (IECBES); 2022. p. 207–212.

[87] Anaam A, Bu-Omer HM, Gofuku A. Studying the Applicability of Generative Adversarial Networks on HEp-2 Cell Image Augmentation. IEEE access : practical innovations, open solutions. 2021;9:98048–98059. doi:10.1109/ACCESS.2021.3095391.

[88] Dimitrakopoulos P, Sfikas G, Nikou C. ISING-GAN: Annotated Data Augmentation with a Spatially Constrained Generative Adversarial Network. In: 2020 IEEE 17th International Symposium on Biomedical Imaging (ISBI); 2020. p. 1600–1603.

[89] Caicedo JC, Goodman A, Karhohs KW, Cimini BA, Ackerman J, Haghighi M, et al. Nucleus Segmentation across Imaging Experiments: The 2018 Data Science Bowl. Nature Methods. 2019;16(12):1247–1253. doi:10.1038/s41592-019-0612-7.

[90] Verma R, Mehrotra R, Rane C, Tiwari R, Agariya AK. Synthetic Image Augmentation with Generative Adversarial Network for Enhanced Performance in Protein Classification. Biomedical Engineering Letters. 2020;10(3):443–452. doi:10.1007/s13534-020-00162-9.

[91] Human Protein Atlas - Single Cell Classification;.

[92] Hussain S, Anees A, Das A, Nguyen BP, Marzuki M, Lin S, et al. High-Content Image Generation for Drug Discovery Using Generative Adversarial Networks. Neural Networks. 2020;132:353–363. doi:10.1016/j.neunet.2020.09.007.

[93] Kastaniotis D, Ntinou I, Tsourounis D, Economou G, Fotopoulos S. Attention-Aware Generative Adversarial Networks (ATA-GANs). In: 2018 IEEE 13th Image, Video, and Multidimensional Signal Processing Workshop (IVMSP); 2018. p. 1–5.

[94] Percannella G, Foggia P, Soda P. HEp-2 Cells Classification Contest. In: 21st International Conference on Pattern Recognition (ICPR); 2012.

[95] Osokin A, Chessel A, Salas REC, Vaggi F. GANs for Biological Image Synthesis. In: 2017 IEEE International Conference on Computer Vision (ICCV); 2017. p. 2252–2261.

[96] Dodgson J, Chessel A, Vaggi F, Giordan M, Yamamoto M, Arai K, et al.. Reconstructing Regulatory Pathways by Systematically Mapping Protein Localization Interdependency Networks; 2017.

[97] Shaga Devan K, Walther P, von Einem J, Ropinski T, A Kestler H, Read C. Improved Automatic Detection of Herpesvirus Secondary Envelopment Stages in Electron Microscopy by Augmenting Training Data with Synthetic Labelled Images Generated by a Generative Adversarial Network. Cellular Microbiology. 2021;23(2):e13280. doi:10.1111/cmi.13280.

[98] Shaham TR, Dekel T, Michaeli T. SinGAN: Learning a Generative Model From a Single Natural Image. In: Proceedings of the IEEE/CVF International Conference on Computer Vision. IEEE Computer Society; 2019. p. 4570–4580.

[99] Han L, Murphy RF, Ramanan D. Learning Generative Models of Tissue Organization with Supervised GANs. In: IEEE Winter Conference on Applications of Computer Vision. IEEE Winter Conference on Applications of Computer Vision. vol. 2018; 2018. p. 682–690.

[100] Zhao T, Murphy RF. Automated Learning of Generative Models for Subcellular Location: Building Blocks for Systems Biology. Cytometry Part A. 2007;71A(12):978–990. doi:10.1002/cyto.a.20487.

[101] Gerhard S, Funke J, Martel J, Cardona A, Fetter R. Segmented Anisotropic ssTEM Dataset of Neural Tissue; 2013.

[102] Rubin M, Stein O, Turko NA, Nygate Y, Roitshtain D, Karako L, et al. TOP-GAN: Stain-free Cancer Cell Classification Using Deep Learning with a Small Training Set. Medical Image Analysis. 2019;57:176–185. doi:10.1016/j.media.2019.06.014.
