# Supplementary material for: Systematic review of generative adversarial networks (GANs) in cell microscopy: Trends, practices, and impact on image augmentation
Source: PLoS One. 2025 Jun 24;20(6):e0291217. doi: 10.1371/journal.pone.0291217 (PMC12186945; doi:10.1371/journal.pone.0291217)
Supplement: S2 Table — (PDF) [file pone.0291217.s002.pdf]

S2 Table. Cell microscopy datasets used in the studies meeting the eligibility criteria, full version.

Dataset	Modality	Number of samples	Resolution	Original Task	Other applications	Annotated	Cell type	URL
RxRx19a Sars-CoV-2 image collection [1] (2020)	Fluorescence microscopy	$305,520$	$1,024 \times 1,024 \times 5$	General purpose dataset	Image augmentation, Feature extraction [2], Transfer learning [3], Treatment assessment [4].	No	HRCE, Vero	`https://www.rxrx.ai/rxrx19`
smFISH dataset [5] [6] [7] (2020)	Fluorescence microscopy	99	$1,024 \times 1,024$	RNA expression study	Image augmentation	Yes	MCF7	`https://data.mendeley.com/datasets/6hsf4fyhsn/2` `https://data.mendeley.com/datasets/9s9m4wytfw/1` `https://data.mendeley.com/datasets/cv7n2bbcb4/1`
RxRx1 dataset [8] (2019)	Fluorescence microscopy	$125,510$	$512 \times 512 \times 6$	Classification	Image augmentation, Transfer learning [3], Domain shift adaptation [9],Transfer learning [4].	Yes	HUVEC, RPE, U2OS, HepG2	`https://www.rxrx.ai/rxrx1`
Human protein atlas image classification Kaggle competition [10] (2019)	Fluorescence microscopy	$31,072$	512×512 or mix between $2,048 \times 2,048$ and $3,072 \times 3,072$	Classification	Image augmentation, Super-resolution [11].	Yes	Human cell	`https://www.kaggle.com/competitions/human-protein-atlas-image-classification/`
LIN dataset [12] (2017)	Fluorescence microscopy	$170,000$	80×48 or 160×96	Identification (protein interactions)	Image augmentation	Yes	Yeast	`https://github.com/aosokin/biogans`
Salivary Gland Tumor and Fallopian datasets [13] (2023)	Immunofluorescence microscopy	40	Variable resolution	Segmentation	Image augmentation	Yes	Fallopian tube and Salivary gland tumor	`https://zenodo.org/records/8096773`

Dataset	Modality	Number of samples	Resolution	Original Task	Other applications	Annotated	Cell type	URL
I3A dataset [14] (2016)	Immunofluorescence microscopy	$13,596$	Unspecified	Classification	Image augmentation, Segmentation [15].	Yes	Hep-2 cell line	`https://hep2.unisa.it/dbtools.html`
Arabidopsis thaliana dataset [16] (2016)	Confocal fluorescence microscopy	125	Between $326\times367\times107$ and $512\times512\times396$	Kinetics analysis	Image augmentation, Segmentation [17]	Yes	*Arabidopsis thaliana*	`https://www.repository.cam.ac.uk/handle/1810/262530`
BioEmergences datasets [18] (2016)	Fluorescence microscopy	394	512×512 with slices from 104 to 120	Preprocessing and cell linage reconstruction	Image augmentation	No	*Danio rerio*	`http://bioemergences.iscpif.fr/bioemergences/openworkflow-datasets.php`
Hep-2 Cells Classification Contest [19] (2013)	Fluorescence microscopy	28	$1,388\times1,038$	Classification contest	Image augmentation, Segmentation [20], Cell count [21].	Yes	Hep-2 cell line	`https://mivia.unisa.it/datasets/biomedical-image-datasets/hep2-image-dataset/`
BBBC038v1 [22] (2012)	Fluorescence microscopy, Optical microscopy	670	From 256×256 to $1,040\times1,388$	Identification and segmentation	Image augmentation, Performance metric assessment [23]	Yes	Different organisms including human, mice and flies	`https://bbbc.broadinstitute.org/BBBC038`
Barrera et al. dataset [24] (2024)	Optical microscopy	$5,605$	360×363	Classification	Image augmentation	Yes	neutrophils	`https://data.mendeley.com/datasets/rh3jw43hjs/1`
Ghose et al. dataset [25] (2023)	Optical microscopy	Unspecified	$7,000\times6,000$ approx.	Classification	Image augmentation	Yes	Human cell	Accessible under request
Raabin WBC [26] (2022)	Optical microscopy	$20,936$	Unspecified	Classification and Segmentation	Image augmentation	Yes	Blood cells	`https://raabindata.com/free-data/`
Asthma Equidae dataset [27] (2021)	Optical microscopy	6	Unspecified	Object detection	Image augmentation	Partially	Equine blood cells	Accessible under request
Tharun and Thompson dataset [28] (2021)	Optical microscopy	156	Unspecified	Classification	Image augmentation	Yes	Thyroid gland tumor cells	Available under request to `sekretariat.patho@uksh.de`

Dataset	Modality	Number of samples	Resolution	Original Task	Other applications	Annotated	Cell type	URL
PBC dataset [29] (2020)	Optical microscopy	$17,092$	360×363	Classification	Image augmentation, Segmentation [30]	Yes	Peripheral Blood cells	`https://data.mendeley.com/datasets/snkd93bnjr/1`
Liquid-based cytology Pap smear dataset [31] (2019)	Optical microscopy	963	$2,048 \times 1,536$	Classification and Segmentation	Image augmentation	Yes	Skin cancer	`https://data.mendeley.com/datasets/zddtpgzv63/4`
SIPaKMeD dataset [32] (2018)	Optical microscopy	$4,049$	$2,048 \times 1,536$	Classification	Image Augmentation, Domain adaptation [33], Segmentation [34]	Yes	Pap-smear	`https://www.cs.uoi.gr/~marina/sipakmed.html`
NCT-CRC-HE-100K dataset [35] (2018)	Optical microscopy	$100,000$	224×224	Classification	Image Augmentation, RNA expression prediction [36], Domain adaptation [37]	Yes	Human colorectal cancer	`https://zenodo.org/records/1214456`
BCCD dataset [38] (2017)	Optical microscopy	365	640×480	Cell detection	Image augmentation, Object classification [39], Segmentation [40].	Yes	Blood cells	`https://github.com/Shenggan/BCCD_Dataset`
BreakHis dataset [41] (2016)	Optical microscopy	$9,109$	Variable resolution	Classification	Image augmentation [42], Segmentation [43], Domain adaptation [44]	Yes	Breast cancer cells	`https://web.inf.ufpr.br/vri/databases/breast-cancer-histopathological-database-breakhis/`
Nikiforov dataset (2016)	Optical microscopy	278	Variable resolution	Nomenclature revision	Image augmentation, Classification [45]	Yes	Encapsulated follicular variant papillary thyroid carcinoma	`https://image.upmc.edu/NikiForov%20EFV%20Study/view.apml`
BM dataset [46] (2015)	Optical microscopy	11	$1,200 \times 1,200$	Cell detection	Image augmentation, Cell segmentation [47], Cell count [48], I2I translation [49].	Yes	Healthy human bone marrow	`https://github.com/pkainz/MICCAI2015/`

**Dataset**	**Modality**	**Number of samples**	**Resolution**	**Original Task**	**Other applications**	**Annotated**	**Cell type**	**URL**
Vancouver General Hospital cohort [50] (2011)	Optical microscopy	$1,286$	$1,128 \times 720$	Prediction (cancer prognosis), classification, and segmentation	Image augmentation, Color pre-processing assessment [51].	Yes	Breast cancer cells	`https://tma.im/tma_portal/C-Path/supp.html`
Pap-smear image dataset [52] (2005)	Optical microscopy	917	Variable resolution (single-cell dataset)	Diagnose	Image augmentation, Classification [53], Cell segmentation [54].	Yes	Pap-smear	`http://mde-lab.aegean.gr/index.php/downloads`
HCMV dataset [55] (2021)	Transmission electron microscopy	350	$2,048 \times 2,048$	Object detection	Image augmentation	Yes	*Herpesviridae*, human *cytomegalovirus*	Accessible under request
VNC dataset [56] (2013)	Transmission Electron Microscopy	40	$1,024 \times 1,024$	Segmentation	Image augmentation, Domain adaptation [57], Quantitative imaging [58].	Yes	*Drosophila melanogaster* instar larva ventral nerve cord	`https://figshare.com/articles/dataset/Segmented_anisotropic_ssTEM_dataset_of_neural_tissue/856713`
TCGA-CRC-DX [59]	Optical Microscopy	$51,918$	512×512	Classification	Image augmentation	Yes	Colorectal Cancer	`https://zenodo.org/records/3832231`

References

[1] RxRx19a Dataset;. https://www.rxrx.ai/rxrx19a.

[2] Heiser K, McLean PF, Davis CT, Fogelson B, Gordon HB, Jacobson P, et al.. Identification of Potential Treatments for COVID-19 through Artificial Intelligence-Enabled Phenomic Analysis of Human Cells Infected with SARS-CoV-2; 2020.

[3] Hua SBZ, Lu AX, Moses AM. CytoImageNet: A Large-Scale Pretraining Dataset for Bioimage Transfer Learning; 2021.

[4] Zhuang D, Ibrahim AK. Deep Learning for Drug Discovery: A Study of Identifying High Efficacy Drug Compounds Using a Cascade Transfer Learning Approach. Applied Sciences. 2021;11(17):7772. doi:10.3390/app11177772.

[5] Zuckerman B, Ulitsky I. smFISH Data for Mol. Cell: NORAD and NXF1 Protein Co-Staining; 2020.

[6] Zuckerman B, Ulitsky I. smFISH Data for Mol. Cell: NORAD, MALAT1 and Oligo-dT Probes; siALY+UAP56; 2020.

[7] Zuckerman B, Ulitsky I. smFISH Data for Mol. Cell: NORAD, MALAT1 and Oligo-dT Probes; siNXF1; 2020.

[8] RxRx1 Dataset;. https://www.rxrx.ai/rxrx1.

[9] Zhang M, Marklund H, Dhawan N, Gupta A, Levine S, Finn C. Adaptive Risk Minimization: Learning to Adapt to Domain Shift. In: Ranzato M, Beygelzimer A, Dauphin Y, Liang PS, Vaughan JW, editors. Advances in Neural Information Processing Systems. vol. 34. Curran Associates, Inc.; 2021. p. 23664–23678.

[10] Human Protein Atlas - Single Cell Classification;.

[11] Bhatia V, Kumar Y. Attaining Real-Time Super-Resolution for Microscopic Images Using GAN; 2020.

[12] Dodgson J, Chessel A, Vaggi F, Giordan M, Yamamoto M, Arai K, et al.. Reconstructing Regulatory Pathways by Systematically Mapping Protein Localization Interdependency Networks; 2017.

[13] Tasnadi E, Sliz-Nagy A, Horvath P. Structure Preserving Adversarial Generation of Labeled Training Samples for Single Cell Segmentation; 2023.

[14] Hobson P, Percannella G, Vento M, Wiliem A. Competition on Cells Classification by Fluorescent Image Analysis. In: 2013 IEEE International Conference on Image Processing; 2013.

[15] Li Y, Shen L. cC-GAN: A Robust Transfer-Learning Framework for HEp-2 Specimen Image Segmentation. IEEE Access. 2018;6:14048–14058. doi:10.1109/ACCESS.2018.2808938.

[16] Willis L, Refahi Y, Wightman R, Landrein B, Teles J, Huang KC, et al. Cell Size and Growth Regulation in the Arabidopsis Thaliana Apical Stem Cell Niche. Proceedings of the National Academy of Sciences. 2016;113(51):E8238–E8246. doi:10.1073/pnas.1616768113.

[17] Wang A, Zhang Q, Han Y, Megason S, Hormoz S, Mosaliganti KR, et al. A Novel Deep Learning-Based 3D Cell Segmentation Framework for Future Image-Based Disease Detection. Scientific Reports. 2022;12(1):342. doi:10.1038/s41598-021-04048-3.

[18] Faure E, Savy T, Rizzi B, Melani C, Stašová O, Fabrèges D, et al. A Workflow to Process 3D+time Microscopy Images of Developing Organisms and Reconstruct Their Cell Lineage. Nature Communications. 2016;7(1):8674. doi:10.1038/ncomms9674.

[19] Percannella G, Foggia P, Soda P. HEp-2 Cells Classification Contest. In: 21st International Conference on Pattern Recognition (ICPR); 2012.

[20] Banerjee A, Maji P. A Spatially Constrained Probabilistic Model for Robust Image Segmentation. IEEE Transactions on Image Processing. 2020;29:4898–4910. doi:10.1109/TIP.2020.2975717.

[21] Riccio D, Brancati N, Frucci M, Gragnaniello D. A New Unsupervised Approach for Segmenting and Counting Cells in High-Throughput Microscopy Image Sets. IEEE Journal of Biomedical and Health Informatics. 2019;23(1):437–448. doi:10.1109/JBHI.2018.2817485.

[22] Caicedo JC, Goodman A, Karhohs KW, Cimini BA, Ackerman J, Haghighi M, et al. Nucleus Segmentation across Imaging Experiments: The 2018 Data Science Bowl. Nature Methods. 2019;16(12):1247–1253. doi:10.1038/s41592-019-0612-7.

[23] Dimitrakopoulos P, Sfikas G, Nikou C. Wind: Wasserstein Inception Distance For Evaluating Generative Adversarial Network Performance. In: ICASSP 2020 - 2020 IEEE International Conference on Acoustics, Speech and Signal Processing (ICASSP); 2020. p. 3182–3186.

[24] Barrera Llanga KI, Rodellar J, Alferez S, Merino A. A Deep Learning Approach for Automatic Recognition of Abnormalities in the Cytoplasm of Neutrophils - Dataset; 2024.

[25] Ghose S, Cho S, Ginty F, McDonough E, Davis C, Zhang Z, et al. Predicting Breast Cancer Events in Ductal Carcinoma In Situ (DCIS) Using Generative Adversarial Network Augmented Deep Learning Model. Cancers. 2023;15(7):1922. doi:10.3390/cancers15071922.

[26] Kouzehkanan ZM, Saghari S, Tavakoli S, Rostami P, Abaszadeh M, Mirzadeh F, et al. A Large Dataset of White Blood Cells Containing Cell Locations and Types, along with Segmented Nuclei and Cytoplasm. Scientific Reports. 2022;12(1):1123. doi:10.1038/s41598-021-04426-x.

[27] Marzahl C, Bertram CA, Wilm F, Voigt J, Barton AK, Klopfleisch R, et al. Cell Detection for Asthma on Partially Annotated Whole Slide Images. In: Palm C, Deserno TM, Handels H, Maier A, Maier-Hein K, Tolxdorff T, editors. Bildverarbeitung für die Medizin 2021. Wiesbaden: Springer Fachmedien; 2021. p. 147–152.

[28] Böhland M, Tharun L, Scherr T, Mikut R, Hagenmeyer V, Thompson LDR, et al. Machine Learning Methods for Automated Classification of Tumors with Papillary Thyroid Carcinoma-like Nuclei: A Quantitative Analysis. PLOS ONE. 2021;16(9):1–21. doi:10.1371/journal.pone.0257635.

[29] Acevedo A, Merino A, Alférez S, Molina Á, Boldú L, Rodellar J. A Dataset of Microscopic Peripheral Blood Cell Images for Development of Automatic Recognition Systems. Data in Brief. 2020;30:105474. doi:10.1016/j.dib.2020.105474.

[30] Ferreira FRT, do Couto LM. Using Deep Learning on Microscopic Images for White Blood Cell Detection and Segmentation to Assist in Leukemia Diagnosis. Journal of Supercomputing. 2025;81(2). doi:10.1007/s11227-024-06903-2.

[31] Hussain E, Mahanta LB, Borah H, Das CR. Liquid Based-Cytology Pap Smear Dataset for Automated Multi-Class Diagnosis of Pre-Cancerous and Cervical Cancer Lesions. Data in Brief. 2020;30:105589. doi:10.1016/j.dib.2020.105589.

[32] Plissiti ME, Dimitrakopoulos P, Sfikas G, Nikou C, Krikoni O, Charchanti A. Sipakmed: A New Dataset for Feature and Image Based Classification of Normal and Pathological Cervical Cells in Pap Smear Images. In: 2018 25th IEEE International Conference on Image Processing (ICIP); 2018. p. 3144–3148.

[33] Pan Q, Xue Y, Yang B. A Deformable-Based Source-Free Unsupervised Domain Adaptation Method for Cervical Cell Detection. In: ICASSP 2025 - 2025 IEEE International Conference on Acoustics, Speech and Signal Processing (ICASSP); 2025. p. 1–5.

[34] Lavntaniti K, Plissiti ME, Vrigkas M, Nikou C. Accurate Cell Segmentation Based on Generative Adversarial Networks and Nuclei Guide Factors. In: 2024 IEEE International Symposium on Biomedical Imaging (ISBI). IEEE; 2024. p. 1–4.

[35] Kather JN, Halama N, Marx A. 100,000 Histological Images of Human Colorectal Cancer and Healthy Tissue;.

[36] Schmauch B, Romagnoni A, Pronier E, Saillard C, Maillé P, Calderaro J, et al. A Deep Learning Model to Predict RNA-Seq Expression of Tumours from Whole Slide Images. Nature Communications. 2020;11(1):3877. doi:10.1038/s41467-020-17678-4.

[37] Shen Y, Ke J. Staindiff: Transfer Stain Styles of Histology Images with Denoising Diffusion Probabilistic Models and Self-Ensemble. In: International Conference on Medical Image Computing and Computer-Assisted Intervention. Springer; 2023. p. 549–559.

[38] shenggan. BCCD Dataset; 2017.

[39] Sharma M, Bhave A, Janghel RR. White Blood Cell Classification Using Convolutional Neural Network. In: Wang J, Reddy GRM, Prasad VK, Reddy VS, editors. Soft Computing and Signal Processing. Singapore: Springer; 2019. p. 135–143.

[40] Tavakoli S, Ghaffari A, Kouzehkanan ZM, Hosseini R. New Segmentation and Feature Extraction Algorithm for Classification of White Blood Cells in Peripheral Smear Images. Scientific Reports. 2021;11(1):19428. doi:10.1038/s41598-021-98599-0.

[41] Spanhol FA, Oliveira LS, Petitjean C, Heutte L. A Dataset for Breast Cancer Histopathological Image Classification. IEEE Transactions on Biomedical Engineering. 2016;63(7):1455–1462. doi:10.1109/TBME.2015.2496264.

[42] Yuan L, Rahaman M, Sun H, Li C, Gu Y, Jiang T, et al. A GAN-based Data Augmentation Method for Mitigating Class Imbalance Problem in Histopathological Image Classification. In: 2024 IEEE International Conference on Bioinformatics and Biomedicine (BIBM). IEEE; 2024. p. 5327–5334.

[43] Anu KV, Krishna S, Paulose RR. Computer-Aided Detection of Nuclei from Histopathology Image: Evaluating the Effectiveness of u-Net and Modified Networks for Nuclei Segmentation. In: 2023 3rd International Conference on Intelligent Technologies (CONIT). IEEE; 2023. p. 1–8.

[44] Chaddad A, Wu Y. Enhancing Classification Tasks through Domain Adaptation Strategies. In: 2023 IEEE International Conference on Bioinformatics and Biomedicine (BIBM). IEEE; 2023. p. 1832–1835.

[45] Do TH, Khanh HN. Supporting Thyroid Cancer Diagnosis Based on Cell Classification over Microscopic Images. In: 2022 International Conference on Multimedia Analysis and Pattern Recognition (MAPR); 2022. p. 1–5.

[46] Kainz P, Urschler M, Schulter S, Wohlhart P, Lepetit V. You Should Use Regression to Detect Cells. In: Navab N, Hornegger J, Wells WM, Frangi AF, editors. Medical Image Computing and Computer-Assisted Intervention – MICCAI 2015. Cham: Springer International Publishing; 2015. p. 276–283.

[47] Akram SU, Kannala J, Eklund L, Heikkilä J. Cell Segmentation Proposal Network for Microscopy Image Analysis. In: Carneiro G, Mateus D, Peter L, Bradley A, Tavares JMRS, Belagiannis V, et al., editors. Deep Learning and Data Labeling for Medical Applications. Cham: Springer International Publishing; 2016. p. 21–29.

[48] Ciampi L, Carrara F, Totaro V, Mazziotti R, Lupori L, Santiago C, et al. Learning to Count Biological Structures with Raters' Uncertainty. Medical Image Analysis. 2022;80:102500. doi:10.1016/j.media.2022.102500.

[49] Xing F, Cornish TC. Low-Resource Adversarial Domain Adaptation for Cross-modality Nucleus Detection. In: Wang L, Dou Q, Fletcher PT, Speidel S, Li S, editors. Medical Image Computing and Computer Assisted Intervention – MICCAI 2022. Cham: Springer Nature Switzerland; 2022. p. 639–649.

[50] Hitchcock CL. The Future of Telepathology for the Developing World. Archives of Pathology & Laboratory Medicine. 2011;135(2):211–214. doi:10.5858/135.2.211.

[51] Bianconi F, Kather JN, Reyes-Aldasoro CC. Evaluation of Colour Pre-processing on Patch-Based Classification of H&E-Stained Images. In: Reyes-Aldasoro CC, Janowczyk A, Veta M, Bankhead P, Sirinukunwattana K, editors. Digital Pathology. Cham: Springer International Publishing; 2019. p. 56–64.

[52] Jantzen J, Norup J, Dounias G, Bjerregaard B. Pap-Smear Benchmark Data for Pattern Classification. In: Proc. NiSIS 2005. NiSIS; 2005. p. 1–9.

[53] Zhang L, Lu L, Nogues I, Summers RM, Liu S, Yao J. DeepPap: Deep Convolutional Networks for Cervical Cell Classification. IEEE Journal of Biomedical and Health Informatics. 2017;21(6):1633–1643. doi:10.1109/JBHI.2017.2705583.

[54] Zhang L, Sonka M, Lu L, Summers RM, Yao J. Combining Fully Convolutional Networks and Graph-Based Approach for Automated Segmentation of Cervical Cell Nuclei. In: 2017 IEEE 14th International Symposium on Biomedical Imaging (ISBI 2017); 2017. p. 406–409.

[55] Shaga Devan K, Walther P, von Einem J, Ropinski T, A Kestler H, Read C. Improved Automatic Detection of Herpesvirus Secondary Envelopment Stages in Electron Microscopy by Augmenting Training Data with Synthetic Labelled Images Generated by a Generative Adversarial Network. Cellular Microbiology. 2021;23(2):e13280. doi:10.1111/cmi.13280.

[56] Gerhard S, Funke J, Martel J, Cardona A, Fetter R. Segmented Anisotropic ssTEM Dataset of Neural Tissue; 2013.

[57] Liu D, Zhang D, Song Y, Zhang F, O'Donnell L, Huang H, et al. PDAM: A Panoptic-Level Feature Alignment Framework for Unsupervised Domain Adaptive Instance Segmentation in Microscopy Images. IEEE Transactions on Medical Imaging. 2021;40(1):154–165. doi:10.1109/TMI.2020.3023466.

[58] Midtvedt B, Helgadottir S, Argun A, Pineda J, Midtvedt D, Volpe G. Quantitative Digital Microscopy with Deep Learning. Applied Physics Reviews. 2021;8(1):011310. doi:10.1063/5.0034891.

[59] Kather JN. Histological Image Tiles for TCGA-CRC-DX, Color-Normalized, Sorted by MSI Status, Train/Test Split; 2020.
