# Supplementary material for: Systematic review of generative adversarial networks (GANs) in cell microscopy: Trends, practices, and impact on image augmentation
Source: PLoS One. 2025 Jun 24;20(6):e0291217. doi: 10.1371/journal.pone.0291217 (PMC12186945; doi:10.1371/journal.pone.0291217)
Supplement: S3 Table — Complete implementation. (PDF) [file pone.0291217.s003.pdf]

S3 Table. Risk of bias with ROBIS. Complete implementation.

**DOMAIN 1**	**STUDY ELIGIBILITY CRITERIA**
Did the review adhere to pre-defined objectives and eligibility criteria?	Yes
Were the eligibility criteria appropriate for the review question?	Probably yes
Were eligibility criteria unambiguous?	Yes
Were any restrictions in eligibility criteria based on study characteristics appropriate (e.g. date, sample size, study quality, outcomes measured)?	Probably yes
Were any restrictions in eligibility criteria based on sources of information appropriate (e.g. publication status or format, language, availability of data)?	Probably yes
**Concerns regarding specification of study eligibility criteria**	Low
**Rationale for concern:**	All signaling questions were answered as "Yes" or "Probably Yes", so no potential concerns about the specification of eligibility criteria were identified.

**DOMAIN 2**	**IDENTIFICATION AND SELECTION OF STUDIES**
Did the search include an appropriate range of databases/electronic sources for published and unpublished reports?	Probably yes
Were methods additional to database searching used to identify relevant reports?	No
Were the terms and structure of the search strategy likely to retrieve as many eligible studies as possible?	Probably yes
Were restrictions based on date, publication format, or language appropriate?	No
Were efforts made to minimise error in selection of studies?	No
**Concerns regarding methods used to identify and/or select studies**	High
**Rationale for concern:**	Some eligible studies are likely to be missing from the review since no additional searching was performed beyond databases. Moreover, only a single person was in charge of screening title, abstract and whole body to classify a study as legible. Finally, only papers written in English were considered.

**DOMAIN 3**	**DATA COLLECTION AND STUDY APPRAISAL**
Were efforts made to minimise error in data collection?	No
Were sufficient study characteristics available for both review authors and readers to be able to interpret the results?	Probably yes
Were all relevant study results collected for use in the synthesis?	Yes
Was risk of bias (or methodological quality) formally assessed using appropriate criteria?	No
Were efforts made to minimise error in risk of bias assessment?	No
**Concerns regarding methods used to collect data and appraise studies**	High
**Rationale for concern:**	Some bias may have been introduced since only a single person was in charge of data collection, and the nature of the studies do not allow a suitable risk of bias assessment.

DOMAIN 4	SYNTHESIS AND FINDINGS
Did the synthesis include all studies that it should?	Yes
Were all pre-defined analyses reported or departures explained?	No
Was the synthesis appropriate given the nature and similarity in the research questions, study designs and outcomes across included studies?	Probably yes
Was between-study variation (heterogeneity) minimal or addressed in the synthesis?	No information
Were the findings robust, e.g. as demonstrated through funnel plot or sensitivity analyses?	No information
Were biases in primary studies minimal or addressed in the synthesis?	No
**Concerns regarding the synthesis and findings**	High
**Rationale for concern:**	The synthesis is likely to produce biased results because it was not possible to consider bias between studies, and the nature of the studies do not allow to account for variation between them

Domain	Concern	Rationale for concern
1. Concerns regarding specification of study eligibility criteria	Low	All signaling questions were answered as "Yes" or "Probably Yes", so no potential concerns about the specification of eligibility criteria were identified.
2. Concerns regarding methods used to identify and/or select studies	High	Some eligible studies are likely to be missing from the review since no additional searching was performed beyond databases. Moreover, only a single person was in charge of screening title, abstract and whole body to classify a study as legible. Finally, only papers written in English were considered.
3. Concerns regarding methods used to collect data and appraise studies	High	Some bias may have been introduced since only a single person was in charge of data collection, and the nature of the studies do not allow a suitable risk of bias assessment.
4. Concerns regarding the synthesis and findings	High	The synthesis is likely to produce biased results because it was not possible to consider bias between studies, and the nature of the studies do not allow to account for variation between them

RISK OF BIAS IN THE REVIEW	
Did the interpretation of findings address all of the concerns identified in Domains 1 to 4?	No
Was the relevance of identified studies to the review's research question appropriately considered?	Probably yes
Did the reviewers avoid emphasizing results on the basis of their statistical significance?	No
**Risk of bias in the review**	High
**Rationale for risk:**	The main source of risk of bias is that a single person was in charge of the whole review process. However, there is another source of bias and it is the nature of the studies. To our knowledge there are not available tools or methodologies to assess risk of bias in deep learning studies, and not all of the criteria fit in a systematic review in this field.
