# Supplementary material for: Systematic review of generative adversarial networks (GANs) in cell microscopy: Trends, practices, and impact on image augmentation
Source: PLoS One. 2025 Jun 24;20(6):e0291217. doi: 10.1371/journal.pone.0291217 (PMC12186945; doi:10.1371/journal.pone.0291217)
Supplement: S4 Table — (PDF) [file pone.0291217.s004.pdf]

## S5 Table. List of all the studies retrieved with the search strategy.

| Nr. | Year | Source | DOI | Title | Inclusion | Reason |
|---|---|---|---|---|---|---|
| 1 | 2020 | Science Direct | https://doi.org/10.1016/ j.neunet.2020.09.007 | High-content image generation for drug discovery using generative adversarial networks | **Yes** | N/A |
| 2 | 2022 | Science Direct | https://doi.org/10.1016/ j.powera.2022.100084 | 3D generation and reconstruction of the fuel cell catalyst layer using 2D images based on deep learning | No | No microscopy imaging |
| 3 | 2021 | Science Direct | https://doi.org/10.1016/ j.procs.2021.02.101 | Cells image generation method based on VAE-SGAN | **Yes** | N/A |
| 4 | 2022 | Science Direct | https://doi.org/10.1016/ j.patter.2022.100577 | Single-cell multi-modal GAN reveals spatial patterns in single-cell data from triple-negative breast cancer | No | No microscopy imaging |
| 5 | 2020 | Science Direct | https://doi.org/10.1016/ j.bspc.2019.101782 | An image augmentation approach using two-stage generative adversarial network for nuclei image segmentation | No | No image augmentation |
| 6 | 2022 | Science Direct | https://doi.org/10.1016/ j.cmpb.2021.106578 | Super-resolution reconstruction of pneumocystis carinii pneumonia images based on generative confrontation network | No | No image augmentation |
| 7 | 2020 | Science Direct | https://doi.org/10.1016/ j.ins.2020.05.116 | Improved recurrent generative adversarial networks with regularization techniques and a controllable framework | No | No microscopy imaging |
| 8 | 2022 | Science Direct | https://doi.org/10.1016/ j.compbiomed.2021.105064 | Combating data incompetence in pollen images detection and classification for pollinosis prevention | No | No microscopy imaging |
| 9 | 2022 | Science Direct | https://doi.org/10.1016/ j.bbe.2022.07.003 | Cell image augmentation for classification task using GANs on Pap smear dataset | No | No image augmentation |
| 10 | 2021 | Science Direct | https://doi.org/10.1016/ j.heliyon.2021.e06331 | Mutual stain conversion between Giemsa and Papanicolaou in cytological images using cycle generative adversarial network | No | No image augmentation |
| 11 | 2020 | Science Direct | https://doi.org/10.1016/ j.neucom.2019.12.040 | Stochastic reconstruction of 3D porous media from 2D images using generative adversarial networks | No | No microscopy imaging |
| 12 | 2022 | Science Direct | https://doi.org/10.1016/ j.media.2021.102337 | SAFRON: Stitching Across the Frontier Network for Generating Colorectal Cancer Histology Images | No | No image augmentation |
| 13 | 2021 | Science | https://doi.org/10.1016/ | GiGAN: Gate in GAN, could gate mechanism filter the features in image-to-image | No | No microscopy |

| | | | | | | |
|---|---|---|---|---|---|---|
| | | Direct | j.neucom.2021.07.085 | translation? | | imaging |
| 14 | 2022 | Science Direct | https://doi.org/10.1016/ j.asoc.2021.108156 | Joint segmentation and classification task via adversarial network: Application to HEp-2 cell images | No | No image augmentation |
| 15 | 2022 | Science Direct | https://doi.org/10.1016/ j.petrol.2022.110652 | Seismic inverse modeling method based on generative adversarial networks | No | No microscopy imaging |
| 16 | 2022 | Science Direct | https://doi.org/10.1016/ j.jclepro.2022.134698 | An integrated framework of optimized learning networks for classifying oil-mixed microplastics | No | No microscopy imaging |
| 17 | 2023 | Science Direct | https://doi.org/10.1016/ j.cmpb.2022.107314 | Automatic generation of artificial images of leukocytes and leukemic cells using generative adversarial networks (syntheticcellgan) | **Yes** | N/A |
| 18 | 2023 | Science Direct | https://doi.org/10.1016/ j.microrel.2022.114887 | Small sample classification based on data enhancement and its application in flip chip defection | No | No microscopy imaging |
| 19 | 2023 | Science Direct | https://doi.org/10.1016/ j.bspc.2022.104562 | Generative adversarial feature learning for glomerulopathy histological classification | No | No microscopy imaging |
| 20 | 2023 | Science Direct | https://doi.org/10.1016/ j.compmedimag.2023.102185 | Unpaired virtual histological staining using prior-guided generative adversarial networks | No | No image augmentation |
| 21 | 2023 | Science Direct | https://doi.org/10.1016/ j.jbi.2023.104303 | Explainable synthetic image generation to improve risk assessment of rare pediatric heart transplant rejection | **Yes** | N/A |
| 22 | 2023 | Science Direct | https://doi.org/10.1016/ j.cad.2023.103498 | Reconstruction of 3D Random Media from 2D Images: Generative Adversarial Learning Approach | No | No microscopy imaging |
| 23 | 2023 | Science Direct | https://doi.org/10.1016/ j.media.2023.102768 | Unpaired mesh-to-image translation for 3D fluorescent microscopy images of neurons | No | No image augmentation |
| 24 | 2023 | Science Direct | https://doi.org/10.1016/ j.aca.2023.341129 | Spectral classification by generative adversarial linear discriminant analysis | No | No microscopy imaging |
| 25 | 2023 | Science Direct | https://doi.org/10.2196/47859 | Synthetic Tabular Data Based on Generative Adversarial Networks in Health Care: Generation and Validation Using the Divide-and-Conquer Strategy | No | No microscopy imaging |
| 26 | 2023 | Science Direct | https://doi.org/10.1016/ j.jpowsour.2023.233286 | Time to market reduction for hydrogen fuel cell stacks using Generative Adversarial Networks | No | No microscopy imaging |

| | | | | | | |
|---|---|---|---|---|---|---|
| 27 | 2023 | Science Direct | https://doi.org/10.1016/j.aej.2023.06.062 | DeepLabV3, IBCO-based ALCResNet: A fully automated classification, and grading system for brain tumor | No | No image augmentation |
| 28 | 2023 | Science Direct | https://doi.org/10.1016/j.bspc.2023.105191 | A deep learning self-attention cross residual network with Info-WGANGP for mitotic cell identification in HEp-2 medical microscopic images | **Yes** | N/A |
| 29 | 2023 | Science Direct | https://doi.org/10.1016/j.cmpb.2023.107718 | CervixFormer: A Multi-scale swin transformer-Based cervical pap-Smear WSI classification framework | **Yes** | N/A |
| 30 | 2023 | Science Direct | https://doi.org/10.1016/j.jpowsour.2023.233411 | Conditional generative adversarial network for generation of three-dimensional porous structure of solid oxide fuel cell anodes with controlled volume fractions | No | No microscopy imaging |
| 31 | 2023 | Science Direct | https://doi.org/10.1016/j.media.2023.102961 | Collagen fiber centerline tracking in fibrotic tissue via deep neural networks with variational autoencoder-based synthetic training data generation | No | No microscopy imaging |
| 32 | 2023 | Science Direct | https://doi.org/10.1016/j.crmeth.2023.100592 | Structure preserving adversarial generation of labeled training samples for single-cell segmentation | **Yes** | N/A |
| 33 | 2023 | Science Direct | https://doi.org/10.1016/j.media.2023.102969 | Learning with limited target data to detect cells in cross-modality images | No | No image augmentation |
| 34 | 2023 | Science Direct | https://doi.org/10.1016/j.asoc.2023.110890 | Semi-supervised generative adversarial networks with spatial coevolution for enhanced image generation and classification | No | No microscopy imaging |
| 35 | 2024 | Science Direct | https://doi.org/10.1016/j.media.2023.102995 | SynCLay: Interactive synthesis of histology images from bespoke cellular layouts | No | No image augmentation |
| 36 | 2023 | Science Direct | https://doi.org/10.1021/acs.molpharmaceut.3c00444 | Designing Cell Delivery Peptides and SARS-CoV-2-Targeting Small Interfering RNAs: A Comprehensive Bioinformatics Study with Generative Adversarial Network-Based Peptide Design and In Vitro Assays | No | No microscopy imaging |
| 37 | 2024 | Science Direct | https://doi.org/10.1016/j.neunet.2023.12.030 | Top-down generation of low-resolution representations improves visual perception and imagination | No | No microscopy imaging |
| 38 | 2024 | Science Direct | https://doi.org/10.1016/j.cmpb.2023.107991 | Isotropic multi-scale neuronal reconstruction from high-ratio expansion microscopy with contrastive unsupervised deep generative models | No | No image augmentation |

| | | | | | | |
|---|---|---|---|---|---|---|
| 39 | 2024 | Science Direct | https://doi.org/10.1016/ j.procs.2024.04.209 | Deep Learning Approach for Automated Data Augmentation and Multi-class Classification of Pap Smear Images | **Yes** | N/A |
| 40 | 2024 | Science Direct | https://doi.org/10.1016/ j.compbiomed.2024.108046 | DSFF-GAN: A novel stain transfer network for generating immunohistochemical image of endometrial cancer | No | No image augmentation |
| 41 | 2024 | Science Direct | https://doi.org/10.1016/ j.compbiomed.2024.108146 | Efficient leukocytes detection and classification in microscopic blood images using convolutional neural network coupled with a dual attention network | **Yes** | N/A |
| 42 | 2024 | Science Direct | https://doi.org/10.1016/ j.vlsi.2024.102186 | Qualitative data augmentation for performance prediction in VLSI circuits | No | No microscopy imaging |
| 43 | 2024 | Science Direct | https://doi.org/10.1016/ j.engappai.2024.108221 | Diffusion-based Wasserstein generative adversarial network for blood cell image augmentation | **Yes** | N/A |
| 44 | 2024 | Science Direct | https://doi.org/10.1016/ j.compbiomed.2024.108410 | Using histopathology latent diffusion models as privacy-preserving dataset augmenters improves downstream classification performance | **Yes** | N/A |
| 45 | 2024 | Science Direct | https://doi.org/10.1016/ j.eswa.2024.124230 | An improved hybrid solar cell defect detection approach using Generative Adversarial Networks and weighted classification | No | No microscopy imaging |
| 46 | 2024 | Science Direct | https://doi.org/10.1016/ j.synbio.2024.05.005 | A generative benchmark for evaluating the performance of fluorescent cell image segmentation | **Yes** | N/A |
| 47 | 2024 | Science Direct | https://doi.org/10.1016/ j.compbiomed.2024.108691 | A deep learning approach for automatic recognition of abnormalities in the cytoplasm of neutrophils | **Yes** | N/A |
| 48 | 2024 | Science Direct | https://doi.org/10.1016/ j.neuroimage.2024.120674 | Gray matters: ViT-GAN framework for identifying schizophrenia biomarkers linking structural MRI and functional network connectivity | No | No microscopy imaging |
| 49 | 2024 | Science Direct | https://doi.org/10.1016/ j.advwatres.2024.104748 | Generation of pore-space images using improved pyramid Wasserstein generative adversarial networks | No | No microscopy imaging |
| 50 | 2024 | Science Direct | https://doi.org/10.1016/ j.bspc.2024.106514 | Novel Neural Style Transfer based data synthesis method for phase-contrast wound healing assay images | No | No image augmentation |
| 51 | 2024 | Science Direct | https://doi.org/10.1016/ j.asoc.2024.111819 | Acute leukemia prediction and classification using convolutional neural network and generative adversarial network | No | No microscopy imaging |

| | | | | | | |
|---|---|---|---|---|---|---|
| 52 | 2024 | Science Direct | https://doi.org/10.1016/j.compstruct.2024.118360 | Multi deep learning-based stochastic microstructure reconstruction and high-fidelity micromechanics simulation of time-dependent ceramic matrix composite response | No | No microscopy imaging |
| 53 | 2024 | Science Direct | https://doi.org/10.1016/j.compbiomed.2024.108913 | Improving quantitative prediction of protein subcellular locations in fluorescence images through deep generative models | No | No microscopy imaging |
| 54 | 2024 | Science Direct | https://doi.org/10.1016/j.engappai.2024.108993 | Generative adversarial networks enable outlier detection and property monitoring for additive manufacturing of complex structures | No | No microscopy imaging |
| 55 | 2024 | Science Direct | https://doi.org/10.1016/j.tsep.2024.102816 | Thermal analysis of fuel cells in renewable energy systems using Generative Adversarial Networks (GANs) and Reinforcement learning | No | No microscopy imaging |
| 56 | 2025 | Science Direct | https://doi.org/10.1016/j.apenergy.2024.124385 | A novel state of health estimation method for lithium-ion battery pack based on cross generative adversarial networks | No | No microscopy imaging |
| 57 | 2024 | Science Direct | https://doi.org/10.1016/j.teler.2024.100163Get rights and content | A deep learning approach for white blood cells image generation and classification using SRGAN and VGG19 | No | No image augmentation |
| 58 | 2024 | Science Direct | https://doi.org/10.1016/j.heliyon.2024.e37902 | HcGAN: Harmonic conditional generative adversarial network for efficiently generating high-quality IHC images from H&E | No | No image augmentation |
| 59 | 2024 | Science Direct | https://doi.org/10.1016/j.ijhydene.2024.11.158 | Prediction of electrode microstructure of SOFC with conditional generative adversarial network | No | No microscopy imaging |
| 60 | 2024 | Science Direct | https://doi.org/10.1016/j.compstruct.2024.118814 | Inverse design of triply periodic minimal surfaces structure based on point cloud generation network | No | No microscopy imaging |
| 61 | 2024 | Science Direct | https://doi.org/10.1016/j.bspc.2024.107359 | VGLGAN: Enhancing NIR-IIb image generation with vision transformer empowered global and local feature generative adversarial network | No | No microscopy imaging |
| 62 | 2025 | Science Direct | https://doi.org/10.1016/j.mtcomm.2025.111625 | Artificial data generation: A strategy to improve efficiency predictions in mixed Sn-Pb perovskite solar cells | No | No microscopy imaging |
| 63 | 2025 | Science Direct | https://doi.org/10.1016/j.imavis.2025.105432 | Advancing brain tumor segmentation and grading through integration of FusionNet and IBCO-based ALCResNet | No | No microscopy imaging |
| 64 | 2025 | Science Direct | https://doi.org/10.1016/j.cmpb.2025.108621 | Semantic-driven synthesis of histological images with controllable cellular distributions | No | No image augmentation |

| | | | | | | |
|---|---|---|---|---|---|---|
| 65 | 2025 | Science Direct | https://doi.org/10.1016/j.procs.2025.02.206 | DCGAN-based Cytology Image Augmentation for Cervical Cancer Cell Classification Using Transfer Learning | **Yes** | N/A |
| 66 | 2025 | Science Direct | https://doi.org/10.1016/j.est.2025.116151 | A simulation method for battery packs based on cell cloning techniques | No | No microscopy imaging |
| 67 | 2025 | Science Direct | https://doi.org/10.1016/j.media.2025.103567 | ArtiDiffuser: A unified framework for artifact restoration and synthesis for histology images via counterfactual diffusion model | No | No image augmentation |
| 68 | 2021 | bioRxiv | https://doi.org/10.1101/2021.01.15.426872 | Sampling from Disentangled Representations of Single-Cell Data Using Generative Adversarial Networks | No | No microscopy imaging |
| 69 | 2021 | bioRxiv | https://doi.org/10.1101/2021.06.23.449584 | Multi-dynamic Modelling Reveals Strongly Time-varying Resting fMRI Correlations | No | No microscopy imaging |
| 70 | 2019 | bioRxiv | https://doi.org/10.1101/718148 | DeepHiC: A Generative Adversarial Network for Enhancing Hi-C Data Resolution | No | No microscopy imaging |
| 71 | 2020 | bioRxiv | https://doi.org/10.1101/2020.03.20.001016 | CryoGAN: A New Reconstruction Paradigm for Single-Particle Cryo-EM via Deep Adversarial Learning | No | No microscopy imaging |
| 72 | 2022 | bioRxiv | https://doi.org/10.1101/2022.07.26.501607 | CGAN-Cmap: protein contact map prediction using deep generative adversarial neural networks | No | No microscopy imaging |
| 73 | 2021 | bioRxiv | https://doi.org/10.1101/2021.11.22.469634 | Discovering Novel Antimicrobial Peptides in Generative Adversarial Network | No | No microscopy imaging |
| 74 | 2020 | bioRxiv | https://doi.org/10.1101/2020.12.02.408195 | Deep learning enables rapid and robust analysis of fluorescence lifetime imaging in photon-starved conditions | No | No image augmentation |
| 75 | 2019 | bioRxiv | https://doi.org/10.1101/863977 | MB-GAN: Microbiome Simulation via Generative Adversarial Network | No | No microscopy imaging |
| 76 | 2020 | bioRxiv | https://doi.org/10.1101/2020.03.19.999615 | Automated design and optimization of multitarget schizophrenia drug candidates by deep learning | No | No microscopy imaging |
| 77 | 2019 | bioRxiv | https://doi.org/10.1101/550517 | Dilated Saliency U-Net for White Matter Hyperintensities Segmentation using Irregularity Age Map | No | No microscopy imaging |
| 78 | 2020 | bioRxiv | https://doi.org/10.1101/2020.12.24.424262 | DeepImmuno: Deep learning-empowered prediction and generation of immunogenic peptides for T cell immunity | No | No microscopy imaging |

| | | | | | | |
|---|---|---|---|---|---|---|
| 79 | 2021 | bioRxiv | https://doi.org/10.1101/2021.11.29.470292 | Developing an Antiviral Peptides Predictor with Generative Adversarial Network Data Augmentation | No | No microscopy imaging |
| 80 | 2022 | bioRxiv | 10.1093/bioinformatics/btac427 | ResPAN: a powerful batch correction model for scRNA-seq data through residual adversarial networks | No | No microscopy imaging |
| 81 | 2021 | bioRxiv | https://doi.org/10.1101/2021.03.31.21254733 | OCT2Hist: Non-Invasive Virtual Biopsy Using Optical Coherence Tomography | No | No microscopy imaging |
| 82 | 2020 | bioRxiv | https://doi.org/10.1101/2020.10.09.20210138 | Synthesising artificial patient-level data for Open Science - an evaluation of five methods | No | No microscopy imaging |
| 83 | 2017 | bioRxiv | https://doi.org/10.1101/184259 | ARIGAN: Synthetic Arabidopsis Plants using Generative Adversarial Network | No | No microscopy imaging |
| 84 | 2019 | bioRxiv | https://doi.org/10.1101/563775 | Synthetic Promoter Design in Escherichia coli based on Generative Adversarial Network | No | No microscopy imaging |
| 85 | 2021 | bioRxiv | https://doi.org/10.1101/2021.12.18.471222 | Speech-driven Facial Animations Improve Speech-in-Noise Comprehension of Humans | No | No microscopy imaging |
| 86 | 2020 | bioRxiv | https://doi.org/10.1101/2020.06.26.174474 | Quantitative comparison of principal component analysis and unsupervised deep learning using variational autoencoders for shape analysis of motile cells | No | No image augmentation |
| 87 | 2021 | bioRxiv | https://doi.org/10.1101/2021.05.03.442476 | Evaluating Sample Augmentation in Microarray Datasets with Generative Models: A Comparative Pipeline and Insights in Tuberculosis | No | No microscopy imaging |
| 88 | 2020 | bioRxiv | https://doi.org/10.1101/2020.12.21.423789 | Label2label: Training a neural network to selectively restore cellular structures in fluorescence microscopy | No | No image augmentation |
| 89 | 2021 | bioRxiv | https://doi.org/10.1101/2021.11.08.467823 | PccGEO: prior constraints conditioned genetic elements optimization | No | No microscopy imaging |
| 90 | 2021 | bioRxiv | https://doi.org/10.1101/2021.07.16.452736 | Quantification of Myxococcus xanthus Aggregation and Rippling Behaviors: Deep-learning Transformation of Phase-contrast into Fluorescence Microscopy Images | No | No image augmentation |
| 91 | 2021 | bioRxiv | https://doi.org/10.1101/2021.01.07.425779 | High-fidelity fast volumetric brain MRI using synergistic wave-controlled aliasing in parallel imaging and a hybrid denoising generative adversarial network | No | No microscopy imaging |

| | | | | | | |
|---|---|---|---|---|---|---|
| 92 | 2021 | bioRxiv | https://doi.org/ 10.1101/2021.04.23.441224 | Translation of cellular protein localization by generative adversarial network | No | No image augmentation |
| 93 | 2020 | bioRxiv | https://doi.org/ 10.1101/2020.09.28.20203075 | Conditional Generative Adversarial Networks for Individualized Treatment Effect Estimation and Treatment Selection | No | No microscopy imaging |
| 94 | 2022 | bioRxiv | https://doi.org/ 10.1101/2022.05.24.22274901 | Image-to-image generative adversarial networks for synthesizing perfusion parameter maps from DSC-MR images in cerebrovascular disease | No | No microscopy imaging |
| 95 | 2021 | bioRxiv | https://doi.org/ 10.1101/2021.12.03.470880 | Simultaneous Super-Resolution and Distortion Correction for Single-shot EPI DWI using Deep Learning | No | No microscopy imaging |
| 96 | 2019 | bioRxiv | https://doi.org/ 10.1101/711804 | Contrast invariant tuning in human perception of image content | No | No microscopy imaging |
| 97 | 2022 | bioRxiv | https://doi.org/ 10.1101/2022.09.11.507500 | In silico labeling enables kinetic myelination assay in brightfield | No | No image augmentation |
| 98 | 2022 | bioRxiv | https://doi.org/ 10.1101/2022.04.27.489829 | TopoGAN: unsupervised manifold alignment of single-cell data | No | No microscopy imaging |
| 99 | 2021 | bioRxiv | https://doi.org/ 10.1101/2021.03.13.435251 | Multi-omics Data Integration by Generative Adversarial Network | No | No microscopy imaging |
| 100 | 2022 | bioRxiv | https://doi.org/ 10.1101/2022.03.21.485159 | A Machine Learning Framework to Predict Subcellular Morphology of Endothelial Cells for Digital Twin Generation | No | No image augmentation |
| 101 | 2022 | bioRxiv | https://doi.org/ 10.1101/2022.09.12.506445 | Style Transfer Generative Adversarial Networks to Harmonize Multi-Site MRI to a Single Reference Image to Avoid Over-Correction | No | No microscopy imaging |
| 102 | 2021 | bioRxiv | https://doi.org/ 10.1101/2021.12.29.474407 | Computational analysis of transition temperatures (Tts) of proteins fused to elastin-like polypeptide (ELP): deep fake evaluation of proteins, linkers, and trailers features | No | No microscopy imaging |
| 103 | 2021 | bioRxiv | https://doi.org/ 10.1101/2021.02.01.429065 | Wrinkle force microscopy: a new machine learning based approach to predict cell mechanics from images | No | No image augmentation |
| 104 | 2022 | bioRxiv | https://doi.org/ 10.1101/2022.01.03.474864 | Encoding of speech in convolutional layers and the brain stem based on language experience | No | No microscopy imaging |
| 105 | 2021 | bioRxiv | https://doi.org/ 10.1101/2021.12.21.473705 | Deep Learning-powered Bessel-beam Multi-parametric Photoacoustic Microscopy | No | No microscopy imaging |

| | | | | | | |
|---|---|---|---|---|---|---|
| 106 | 2018 | bioRxiv | https://doi.org/10.1101/460188 | GANai: Standardizing CT Images using Generative Adversarial Network with Alternative Improvement | No | No microscopy imaging |
| 107 | 2021 | bioRxiv | https://doi.org/10.1101/2021.07.26.453862 | Alzheimer's Disease Classification Accuracy is Improved by MRI Harmonization based on Attention-Guided Generative Adversarial Networks | No | No microscopy imaging |
| 108 | 2020 | bioRxiv | https://doi.org/10.1101/2020.06.26.173179 | Inferring cellular trajectories from scRNA-seq using Pseudocell Tracer | No | No microscopy imaging |
| 109 | 2020 | bioRxiv | https://doi.org/10.1101/2020.08.05.237834 | Automatic inference of demographic parameters using Generative Adversarial Networks | No | No microscopy imaging |
| 110 | 2021 | bioRxiv | https://doi.org/10.1101/2021.04.29.441920 | Generating realistic cell samples for gene selection in scRNA-seq data: A novel generative framework | No | No microscopy imaging |
| 111 | 2019 | bioRxiv | https://doi.org/10.1101/662692 | Predicting the Evolution of White Matter Hyperintensities in Brain MRI using Generative Adversarial Networks and Irregularity Map | No | No microscopy imaging |
| 112 | 2021 | bioRxiv | https://doi.org/10.1101/2021.05.18.444641 | Generative adversarial neural networks maintain decoder accuracy during signal disruption in simulated long-term recordings from brain computer interfaces | No | No microscopy imaging |
| 113 | 2020 | bioRxiv | https://doi.org/10.1101/2020.02.24.961714 | HiCSR: a Hi-C super-resolution framework for producing highly realistic contact maps | No | No microscopy imaging |
| 114 | 2020 | bioRxiv | https://doi.org/10.1101/2020.06.12.20129643 | Improving effectiveness of different deep learning-based models for detecting COVID-19 from computed tomography (CT) images | No | No microscopy imaging |
| 115 | 2018 | bioRxiv | https://doi.org/10.1101/226688 | Generative adversarial networks for reconstructing natural images from brain activity | No | No microscopy imaging |
| 116 | 2019 | bioRxiv | https://doi.org/10.1101/743179 | A 3D High Resolution Generative Deep-learning Network for Fluorescence Microscopy Image | No | No image augmentation |
| 117 | 2022 | bioRxiv | https://doi.org/10.1101/2022.06.11.495756 | Thinking like a structural biologist: A pocket-based 3D molecule generative model fueled by electron density | No | No image augmentation |
| 118 | 2022 | bioRxiv | https://doi.org/10.1101/2022.06.27.497752 | Three-dimensional mitochondrial fission, fusion and depolarisation event location prediction for a high throughput analysis of fluorescence microscopy images | No | No image augmentation |

| | | | | | | |
|---|---|---|---|---|---|---|
| 119 | 2018 | bioRxiv | https://doi.org/10.1101/272518 | End-to-end deep image reconstruction from human brain activity | No | No microscopy imaging |
| 120 | 2020 | bioRxiv | https://doi.org/10.1101/2020.11.18.388843 | AMPGAN v2: Machine Learning Guided Design of Antimicrobial Peptides | No | No microscopy imaging |
| 121 | 2020 | bioRxiv | https://doi.org/10.1101/2020.07.01.168849 | Hyperrealistic neural decoding: Reconstructing faces from fMRI activations via the GAN latent space | No | No microscopy imaging |
| 122 | 2020 | bioRxiv | https://doi.org/10.1101/2020.04.28.067231 | Predicting sites of epitranscriptome modifications using unsupervised representation learning based on generative adversarial networks | No | No microscopy imaging |
| 123 | 2019 | bioRxiv | https://doi.org/10.1101/567891 | A Novel Transfer Learning Approach for Toxoplasma Gondii Microscopic Image Recognition by Fuzzy Cycle Generative Adversarial Network | No | No image augmentation |
| 124 | 2020 | bioRxiv | https://doi.org/10.1101/2020.07.22.20159814 | Enhancing MR imaging driven Alzheimer's disease classification performance using generative adversarial learning | No | No microscopy imaging |
| 125 | 2021 | bioRxiv | https://doi.org/10.1101/2021.07.23.453477 | GAN-GMHI: Generative Adversarial Network for high discrimination power in microbiome-based disease prediction | No | No microscopy imaging |
| 126 | 2018 | bioRxiv | https://doi.org/10.1101/309641 | Deep learning achieves super-resolution in fluorescence microscopy | No | No image augmentation |
| 127 | 2021 | bioRxiv | https://doi.org/10.1101/2021.04.27.21256189 | DeepFake electrocardiograms: the key for open science for artificial intelligence in medicine | No | No microscopy imaging |
| 128 | 2021 | bioRxiv | https://doi.org/10.1101/2021.12.02.470663 | Epiphany: predicting Hi-C contact maps from 1D epigenomic signals | No | No microscopy imaging |
| 129 | 2017 | bioRxiv | https://doi.org/10.1101/167916 | Capturing the diversity of biological tuning curves using generative adversarial networks | No | No microscopy imaging |
| 130 | 2020 | bioRxiv | https://doi.org/10.1101/2020.06.09.143297 | SC-GAN: 3D self-attention conditional GAN with spectral normalization for multi-modal neuroimaging synthesis | No | No microscopy imaging |
| 131 | 2019 | bioRxiv | https://doi.org/10.1101/563734 | UDCT: Unsupervised data to content transformation with histogram-matching cycle-consistent generative adversarial networks | No | No microscopy imaging |
| 132 | 2018 | bioRxiv | https://doi.org/10.1101/363481 | Generating Realistic Morphologies of Neurons in Rodent Hippocampus with DCGAN | No | No image augmentation |

| | | | | | | |
|---|---|---|---|---|---|---|
| 133 | 2020 | bioRxiv | https://doi.org/10.1101/2020.10.02.322917 | Leveraging high-throughput screening data and conditional generative adversarial networks to advance predictive toxicology | No | No image augmentation |
| 134 | 2021 | bioRxiv | https://doi.org/10.1101/2021.03.17.435892 | Style Transfer Using Generative Adversarial Networks for Multi-Site MRI Harmonization | No | No microscopy imaging |
| 135 | 2021 | bioRxiv | https://doi.org/10.1101/2021.12.10.472084 | De-novo generation of novel phenotypically active molecules for Chagas disease from biological signatures using AI-driven generative chemistry | No | No microscopy imaging |
| 136 | 2022 | bioRxiv | https://doi.org/10.1101/2022.07.12.499846 | scDREAMER: atlas-level integration of single-cell datasets using deep generative model paired with adversarial classifier | No | No microscopy imaging |
| 137 | 2022 | bioRxiv | https://doi.org/10.1101/2022.07.24.501328 | Digitally Predicting Protein Localization and Manipulating Protein Activity in Fluorescence Images Using Four-dimensional Reslicing GAN | No | No image augmentation |
| 138 | 2020 | bioRxiv | https://doi.org/10.1101/2020.02.24.20027193 | Recovering Mandibular Morphology after Disease with Artificial Intelligence | No | No microscopy imaging |
| 139 | 2020 | bioRxiv | https://doi.org/10.1101/2020.07.10.197087 | GAN-based anomaly detection in multi-modal MRI images | No | No microscopy imaging |
| 140 | 2020 | bioRxiv | https://doi.org/10.1101/2020.02.07.939215 | Batch Equalization with a Generative Adversarial Network | No | No microscopy imaging |
| 141 | 2022 | bioRxiv | https://doi.org/10.1101/2022.06.18.496675 | Direct Generation of Protein Conformational Ensembles via Machine Learning | No | No microscopy imaging |
| 142 | 2019 | bioRxiv | https://doi.org/10.1101/867168 | FUNCTIONAL NETWORK CONNECTIVITY (FNC)-BASED GENERATIVE ADVERSARIAL NETWORK (GAN) AND ITS APPLICATIONS IN CLASSIFICATION OF MENTAL DISORDERS | No | No microscopy imaging |
| 143 | 2020 | bioRxiv | https://doi.org/10.1101/2020.08.20.259598 | Generating hard-to-obtain information from easy-to-obtain information: applications in drug discovery and clinical inference | No | No microscopy imaging |
| 144 | 2020 | bioRxiv | https://doi.org/10.1101/2020.01.10.901876 | An image-computable model of human visual shape similarity | No | No microscopy imaging |
| 145 | 2020 | bioRxiv | https://doi.org/10.1101/2020.04.17.047449 | Network-principled deep generative models for designing drug combinations as graph sets | No | No microscopy imaging |
| 146 | 2021 | bioRxiv | https://doi.org/10.1101/2021.12.09.21267472 | Neural networks for classification and image generation of aging in genetic syndromes | No | No microscopy imaging |

| | | | | | | |
|---|---|---|---|---|---|---|
| 147 | 2021 | bioRxiv | https://doi.org/10.1101/2021.01.12.426430 | Super-Resolution Cryo-EM Maps With 3D Deep Generative Networks | No | No microscopy imaging |
| 148 | 2021 | bioRxiv | https://doi.org/10.1101/2021.02.15.431193 | PandoraGAN: Generating antiviral peptides using Generative Adversarial Network | No | No microscopy imaging |
| 149 | 2021 | bioRxiv | https://doi.org/10.1101/2021.12.09.21267472 | Proof-of-principle neural network models for classification, attribution, creation, style-mixing, and morphing of image data for genetic conditions | No | No microscopy imaging |
| 150 | 2020 | bioRxiv | https://doi.org/10.1101/2020.04.12.024844 | Designing Feature-Controlled Humanoid Antibody Discovery Libraries Using Generative Adversarial Networks | No | No microscopy imaging |
| 151 | 2022 | bioRxiv | https://doi.org/10.1101/2022.06.01.494379 | Improving classification and reconstruction of imagined images from EEG signals | No | No microscopy imaging |
| 152 | 2019 | bioRxiv | https://doi.org/10.1101/544130 | Cellular structure image classification with small targeted training samples | No | No image augmentation |
| 153 | 2021 | bioRxiv | https://doi.org/10.1101/2021.07.19.452964 | Resolution Enhancement with a Task-Assisted GAN to Guide Optical Nanoscopy Image Analysis and Acquisition | No | No microscopy imaging |
| 154 | 2020 | bioRxiv | https://doi.org/10.1101/2020.09.19.20197764 | Graph representation forecasting of patient's medical conditions: towards a digital twin | No | No microscopy imaging |
| 155 | 2022 | bioRxiv | https://doi.org/10.1016/j.compbiomed.2022.105596 | MultiHeadGAN: A Deep Learning Method for Low Contrast Retinal Pigment Epithelium Cells Segmentation in Fluorescent Flatmount Microscopy Images | No | No microscopy imaging |
| 156 | 2021 | bioRxiv | https://doi.org/10.1101/2021.05.26.445797 | Experimentally unsupervised deconvolution for light-sheet microscopy with propagation-invariant beams | No | No microscopy imaging |
| 157 | 2020 | bioRxiv | https://doi.org/10.1101/2020.04.29.066464 | Deep feature extraction of single-cell transcriptomes by generative adversarial network | No | No microscopy imaging |
| 158 | 2022 | bioRxiv | https://doi.org/10.1101/2022.01.22.477329 | D2BGAN: Dual Discriminator Bayesian Generative Adversarial Network for Deformable MR-Ultrasound Registration Applied to Brain Shift compensation | No | No microscopy imaging |
| 159 | 2020 | bioRxiv | https://doi.org/10.1101/2020.07.23.218859 | Reconstructing feedback representations in ventral visual pathway with a generative adversarial autoencoder | No | No microscopy imaging |

| | | | | | | |
|---|---|---|---|---|---|---|
| 160 | 2021 | bioRxiv | https://doi.org/10.1101/2021.12.20.21268090 | HeartNet: Self Multi-Head Attention Mechanism via Convolutional Network with Adversarial Data Synthesis for ECG-based Arrhythmia Classification | No | No microscopy imaging |
| 161 | 2022 | bioRxiv | https://doi.org/10.1101/2022.03.14.484346 | Probabilistic Brain Extraction in MR Images via Conditional Generative Adversarial Networks | No | No microscopy imaging |
| 162 | 2021 | bioRxiv | https://doi.org/10.1101/2021.02.11.21250741 | A Method for Machine Learning Generation of Realistic Synthetic Datasets for Validating Healthcare Applications | No | No microscopy imaging |
| 163 | 2021 | bioRxiv | https://doi.org/10.1101/2021.11.10.467885 | Conditional Generative Modeling for De Novo Protein Design with Hierarchical Functions | No | No microscopy imaging |
| 164 | 2022 | bioRxiv | https://doi.org/10.1101/2022.02.02.22270339 | A Utility-Based Machine Learning-Driven Personalized Lifestyle Recommendation for Cardiovascular Disease Prevention | No | No microscopy imaging |
| 165 | 2020 | bioRxiv | https://doi.org/10.1101/2020.07.19.20157321 | Simulating drug effects on blood glucose laboratory test time series with a conditional WGAN | No | No microscopy imaging |
| 166 | 2020 | bioRxiv | https://doi.org/10.1101/2020.10.27.358101 | Counterfactual Hypothesis Testing of Tumor Microenvironment Scenarios Through Semantic Image Synthesis | No | No microscopy imaging |
| 167 | 2019 | bioRxiv | https://doi.org/10.1101/789719 | Expanding functional protein sequence space using generative adversarial networks | No | No microscopy imaging |
| 168 | 2019 | bioRxiv | https://doi.org/10.1101/836254 | Adversarial generation of gene expression data | No | No microscopy imaging |
| 169 | 2019 | bioRxiv | https://doi.org/10.1101/478982 | Generative adversarial network (GAN) enabled on-chip contact microscopy | No | No image augmentation |
| 170 | 2020 | bioRxiv | https://doi.org/10.1101/2020.10.06.323162 | CancerVar: an Artificial Intelligence empowered platform for clinical interpretation of somatic mutations in cancer | No | No image augmentation |
| 171 | 2020 | bioRxiv | https://doi.org/10.1101/738641 | Automatic Spatial Estimation of White Matter Hyperintensities Evolution in Brain MRI using Disease Evolution Predictor Deep Neural Networks | No | No microscopy imaging |
| 172 | 2021 | bioRxiv | https://doi.org/10.1101/2021.05.21.445196 | AxonDeep: Automated Optic Nerve Axon Segmentation in Mice with Deep Learning | No | No image augmentation |

| | | | | | | |
|---|---|---|---|---|---|---|
| 173 | 2022 | bioRxiv | https://doi.org/10.1101/2022.06.19.22276611 | Deep-Learning-Based Generation of Synthetic High-Resolution MRI from Low-Resolution MRI for Use in Head and Neck Cancer Adaptive Radiotherapy | No | No microscopy imaging |
| 174 | 2020 | bioRxiv | https://doi.org/10.1101/2020.05.18.102814 | Using Conditional Generative Adversarial Networks to Boost the Performance of Machine Learning in Microbiome Datasets | No | No microscopy imaging |
| 175 | 2022 | bioRxiv | https://doi.org/10.1101/2022.08.26.504777 | Using adversarial networks to extend brain computer interface decoding accuracy over time | No | No microscopy imaging |
| 176 | 2022 | bioRxiv | https://doi.org/10.1101/2022.09.17.508145 | This population does not exist: learning the distribution of evolutionary histories with generative adversarial networks | No | No microscopy imaging |
| 177 | 2022 | bioRxiv | https://doi.org/10.1101/2022.09.21.22280206 | Generative Editing via Convolutional Obscuring (GECO): A Generative Adversarial Network for MRI de-artifacting | No | No microscopy imaging |
| 178 | 2022 | bioRxiv | https://doi.org/10.1101/2022.09.23.22280215 | ARCliDS: A Clinical Decision Support System for AI-assisted Decision-Making in Response-Adaptive Radiotherapy | No | No microscopy imaging |
| 179 | 2022 | bioRxiv | https://doi.org/10.1101/2022.10.07.22280776 | Applying GAN-based data augmentation to improve transcriptome-based prognostication in breast cancer | No | No microscopy imaging |
| 180 | 2022 | bioRxiv | https://doi.org/10.1101/2022.10.11.511633 | Physics-based Deep Learning for Imaging Neuronal Activity via Two-photon and Light Field Microscopy | No | No microscopy imaging |
| 181 | 2022 | bioRxiv | https://doi.org/10.1101/2022.10.14.512221 | Synthetic EMG Based on Adversarial Style Transfer can Effectively Attack Biometric-based Personal Identification Models | No | No microscopy imaging |
| 182 | 2022 | bioRxiv | https://doi.org/10.1101/2022.10.23.513389 | scCobra: Contrastive cell embedding learning with domain-adaptation for single-cell data integration | No | No microscopy imaging |
| 183 | 2022 | bioRxiv | https://doi.org/10.1101/2022.11.10.515980 | Highly Realistic Whole Transcriptome Synthesis through Generative Adversarial Networks | No | No microscopy imaging |
| 184 | 2022 | bioRxiv | https://doi.org/10.1101/2022.11.15.516349 | UNSUPERVISED HARMONIZATION OF BRAIN MRI USING 3D CYCLE GANS AND ITS EFFECT ON BRAIN AGE PREDICTION | No | No microscopy imaging |
| 185 | 2022 | bioRxiv | https://doi.org/10.1101/2022.11.19.517154 | Maximum Classifier Discrepancy Generative Adversarial Network for Jointly Harmonizing Scanner Effects and Improving Reproducibility of Downstream Tasks | No | No microscopy imaging |

| | | | | | | |
|---|---|---|---|---|---|---|
| 186 | 2022 | bioRxiv | https://doi.org/10.1101/2022.12.09.519505 | Phylogenetic inference using Generative Adversarial Networks | No | No microscopy imaging |
| 187 | 2022 | bioRxiv | https://doi.org/10.1101/2022.12.17.520847 | De novo protein backbone generation based on diffusion with structured priors and adversarial training | No | No microscopy imaging |
| 188 | 2022 | bioRxiv | https://doi.org/10.1101/2022.08.29.505633 | Small Training Dataset Convolutional Neural Networks for Application Specific Super-Resolution Microscopy | No | No image augmentation |
| 189 | 2022 | bioRxiv | https://doi.org/10.1101/2022.04.27.489829 | scTopoGAN: unsupervised manifold alignment of single-cell data | No | No microscopy imaging |
| 190 | 2023 | bioRxiv | https://doi.org/10.1101/2023.01.30.526198 | Learning to Generate 5' UTR Sequences for Optimized Ribosome Load and Gene Expression | No | No microscopy imaging |
| 191 | 2023 | bioRxiv | https://doi.org/10.1101/2023.02.14.528391 | Generative Molecular Design and Experimental Validation of Selective Histamine H1 Inhibitors | No | No microscopy imaging |
| 192 | 2023 | bioRxiv | https://doi.org/10.1101/2023.02.23.23286367 | Predicting Breast Cancer Events in Ductal Carcinoma In Situ (DCIS) using Generative Adversarial Network Augmented Deep Learning Model | **Yes** | N/A |
| 193 | 2023 | bioRxiv | https://doi.org/10.1101/2023.03.04.531015 | Computational Scoring and Experimental Evaluation of Enzymes Generated by Neural Networks | No | No microscopy imaging |
| 194 | 2023 | bioRxiv | https://doi.org/10.1101/2023.01.17.524156 | Predictive Motor Control Based on a Generative Adversarial Network | No | No microscopy imaging |
| 195 | 2023 | bioRxiv | https://doi.org/10.1101/2023.03.07.531546 | Interpreting Generative Adversarial Networks to Infer Natural Selection from Genetic Data | No | No microscopy imaging |
| 196 | 2023 | bioRxiv | https://doi.org/10.1101/2023.03.06.23285299 | MAUDGAN: Motion Artifact Unsupervised Disentanglement Generative Adversarial Network of Multicenter MRI Data with Different Brain tumors | No | No microscopy imaging |
| 197 | 2023 | bioRxiv | https://doi.org/10.1101/2023.03.25.534230 | AnimalGAN: A Generative Adversarial Network Model Alternative to Animal Studies for Clinical Pathology Assessment | No | No microscopy imaging |
| 198 | 2023 | bioRxiv | https://doi.org/10.1101/2023.04.18.537149 | Inferring parameters of pyramidal neuron excitability in mouse models of Alzheimer's disease using biophysical modeling and deep learning | No | No microscopy imaging |

| | | | | | | |
|---|---|---|---|---|---|---|
| 199 | 2023 | bioRxiv | https://doi.org/ 10.1101/2023.04.20.537642 | GAN-MAT: Generative Adversarial Network-based Microstructural Profile Covariance Analysis Toolbox | No | No microscopy imaging |
| 200 | 2023 | bioRxiv | https://doi.org/ 10.1101/2023.04.16.23288633 | SCGAN: Sparse CounterGAN for Counterfactual Explanations in Breast Cancer Prediction | No | No microscopy imaging |
| 201 | 2023 | bioRxiv | https://doi.org/ 10.1101/2023.04.30.538453 | Segmentation of 3D blood vessel networks using unsupervised deep learning | No | No microscopy imaging |
| 202 | 2023 | bioRxiv | https://doi.org/ 10.1101/2023.05.04.539497 | Protein Engineering for Thermostability through Deep Evolution | No | No microscopy imaging |
| 203 | 2023 | bioRxiv | https://doi.org/ 10.1101/2023.05.04.539501 | Identification of Pain-Associated Effusion-Synovitis from Knee Magnetic Resonance Imaging by Deep Generative Networks | No | No microscopy imaging |
| 204 | 2023 | bioRxiv | https://doi.org/ 10.1101/2023.05.22.541691 | Histopathological Domain Adaptation with Generative Adversarial Networks Bridging the Domain Gap Between Thyroid Cancer Histopathology Datasets | **Yes** | N/A |
| 205 | 2023 | bioRxiv | https://doi.org/ 10.1101/2023.06.20.23291680 | Improving irregular temporal modeling by integrating synthetic data to the electronic medical record using conditional GANs: a case study of fluid overload prediction in the intensive care unit | No | No microscopy imaging |
| 206 | 2023 | bioRxiv | https://doi.org/ 10.1101/2023.06.26.546547 | Scalable Integration of Multiomic Single Cell Data Using Generative Adversarial Networks | No | No microscopy imaging |
| 207 | 2023 | bioRxiv | https://doi.org/ 10.1101/2023.07.14.549043 | Generative and predictive neural networks for the design of functional RNA molecules | No | No microscopy imaging |
| 208 | 2023 | bioRxiv | https://doi.org/ 10.1101/2023.07.25.550225 | GRouNdGAN: GRN-guided simulation of single-cell RNA-seq data using causal generative adversarial networks | No | No microscopy imaging |
| 209 | 2023 | bioRxiv | https://doi.org/ 10.1101/2023.08.22.23294405 | Redefining Hemodynamic Imaging in Stroke: Perfusion Parameter Map Generation from TOF-MRA using Artificial Intelligence | No | No microscopy imaging |
| 210 | 2023 | bioRxiv | https://doi.org/ 10.1101/2023.08.28.555223 | A novel f-divergence based generative adversarial imputation method for scRNA-seq data analysis | No | No microscopy imaging |
| 211 | 2023 | bioRxiv | https://doi.org/ 10.1101/2023.09.11.23295380 | Predictive Modeling of Clinical Trial Outcomes for Novel Drugs using Digital Twin Patient Cohorts and GenerativeAI | No | No microscopy imaging |

| | | | | | | |
|---|---|---|---|---|---|---|
| 212 | 2023 | bioRxiv | https://doi.org/10.1101/2023.09.22.559043 | Cardiac disease diagnosis based on GAN in case of missing data | No | No microscopy imaging |
| 213 | 2023 | bioRxiv | https://doi.org/10.1101/2023.10.17.562789 | Bidirectional Generative Adversarial Representation Learning for Natural Stimulus Synthesis | No | No microscopy imaging |
| 214 | 2023 | bioRxiv | https://doi.org/10.1101/2023.10.16.562598 | Surface Generative Modelling of Neurodevelopmental Trajectories | No | No microscopy imaging |
| 215 | 2023 | bioRxiv | https://doi.org/10.1101/2023.11.01.565168 | Indistinguishable network dynamics can emerge from unalike plasticity rules | No | No microscopy imaging |
| 216 | 2023 | bioRxiv | https://doi.org/10.1101/2023.11.12.565422 | Assessment of an AI virtual staining model performance across same and serial tissue sections using CD3+ T cell ground truth | No | No image augmentation |
| 217 | 2023 | bioRxiv | https://doi.org/10.1101/2023.11.13.23298477 | Adversarial Learning for MRI Reconstruction and Classification of Cognitively Impaired Individuals | No | No microscopy imaging |
| 218 | 2023 | bioRxiv | https://doi.org/10.1101/2023.12.06.23299464 | RCT-Twin-GAN Generates Digital Twins of Randomized Control Trials Adapted to Real-world Patients to Enhance their Inference and Application | No | No microscopy imaging |
| 219 | 2023 | bioRxiv | https://doi.org/10.1101/2023.11.22.568376 | scCross: A Deep Generative Model for Unifying Single-cell Multi-omics with Seamless Integration, Cross-modal Generation, and In-silico Exploration | No | No microscopy imaging |
| 220 | 2023 | bioRxiv | https://doi.org/10.1101/2023.12.16.570150 | High-Activity Enhancer Generation based on Feedback GAN with Domain Constraint and Curriculum Learning | No | No microscopy imaging |
| 221 | 2023 | bioRxiv | https://doi.org/10.1101/2023.12.23.573175 | IST-editing: Infinite spatial transcriptomic editing in a generated gigapixel mouse pup | No | No microscopy imaging |
| 222 | 2023 | bioRxiv | https://doi.org/10.1101/2023.12.28.23300409 | Multi-contrast high-field quality image synthesis for portable low-field MRI using generative adversarial networks and paired data | No | No microscopy imaging |
| 223 | 2024 | bioRxiv | https://doi.org/10.1101/2024.01.06.23300659 | Testing federated analytics across secure data environments using differing statistical approaches on cross-disciplinary data | No | No microscopy imaging |
| 224 | 2024 | bioRxiv | https://doi.org/10.1101/2024.01.11.575307 | Gray Matters: An Efficient Vision Transformer GAN Framework for Predicting Functional Network Connectivity Biomarkers from Brain Structure | No | No microscopy imaging |

| | | | | | | |
|---|---|---|---|---|---|---|
| 225 | 2024 | bioRxiv | https://doi.org/10.1101/2024.01.25.577225 | Synthetic Histology Images for Training AI Models: A Novel Approach to Improve Prostate Cancer Diagnosis | **Yes** | N/A |
| 226 | 2024 | bioRxiv | https://doi.org/10.1101/2024.02.14.580420 | Genome-AC-GAN: Enhancing Synthetic Genotype Generation through Auxiliary Classification | No | No microscopy imaging |
| 227 | 2024 | bioRxiv | https://doi.org/10.1101/2024.02.15.580528 | DiffGAN: a conditional generative adversarial network for phasing single molecule diffraction data to atomic resolution | No | No microscopy imaging |
| 228 | 2023 | bioRxiv | https://doi.org/10.1101/2023.04.30.538453 | Unsupervised segmentation of 3D microvascular photoacoustic images using deep generative learning | No | No microscopy imaging |
| 229 | 2024 | bioRxiv | https://doi.org/10.1101/2024.03.03.581078 | Generative Adversarial Implicit Successor Representation | No | No microscopy imaging |
| 230 | 2024 | bioRxiv | https://doi.org/10.1101/2024.03.02.583135 | DUAL: deep unsupervised simultaneous simulation and denoising for cryo-electron tomography | No | No image augmentation |
| 231 | 2024 | bioRxiv | https://doi.org/10.1101/2024.03.22.586306 | Generative Adversarial Networks Accurately Reconstruct Pan-Cancer Histology from Pathologic, Genomic, and Radiographic Latent Features | **Yes** | N/A |
| 232 | 2024 | bioRxiv | https://doi.org/10.1101/2024.03.23.586408 | Generative Models for Prediction of Non-B DNA Structures | No | No microscopy imaging |
| 233 | 2024 | bioRxiv | https://doi.org/10.1101/2024.04.02.24305197 | Generative Modeling of the Circle of Willis Using 3D-StyleGAN | No | No microscopy imaging |
| 234 | 2024 | bioRxiv | https://doi.org/10.1101/2024.04.12.589174 | Tumor spheroid elasticity estimation using mechano-microscopy combined with a conditional generative adversarial network | No | No image augmentation |
| 235 | 2024 | bioRxiv | https://doi.org/10.1101/2024.04.15.24305876 | VarPPUD: Variant post prioritization developed for undiagnosed genetic disorders | No | No microscopy imaging |
| 236 | 2024 | bioRxiv | https://doi.org/10.1101/2024.02.23.24303275 | Quantitative Characterization of Retinal Features in Translated OCTA | No | No microscopy imaging |
| 237 | 2022 | bioRxiv | https://doi.org/10.1101/2022.10.23.513389 | scCobra: Contrastive cell embedding learning with domain-adaptation for single-cell data integration and harmonization | No | No microscopy imaging |
| 238 | 2024 | bioRxiv | https://doi.org/10.1101/2024.05.12.593773 | Machine Learning Approaches for Skin Neoplasm Diagnosis | No | No microscopy imaging |

| | | | | | | |
|---|---|---|---|---|---|---|
| 239 | 2024 | bioRxiv | https://doi.org/ 10.1101/2024.05.15.594361 | An AI-Driven Framework for Discovery of BACE1 Inhibitors for Alzheimer's Disease | No | No microscopy imaging |
| 240 | 2024 | bioRxiv | https://doi.org/ 10.1101/2024.05.28.24308027 | Enhancing the Diagnostic Utility of ASL Imaging in Temporal Lobe Epilepsy through FlowGAN: An ASL to PET Image Translation Framework | No | No microscopy imaging |
| 241 | 2024 | bioRxiv | https://doi.org/ 10.1101/2024.05.29.24308097 | Generative Artificial Intelligence Model for Simulating Brain Structural Changes in Schizophrenia | No | No microscopy imaging |
| 242 | 2024 | bioRxiv | https://doi.org/ 10.1101/2024.06.01.24308319 | Efficient Synthesis of 3D MR Images for Schizophrenia Diagnosis Classification with Generative Adversarial Networks | No | No microscopy imaging |
| 243 | 2024 | bioRxiv | https://doi.org/ 10.1101/2024.06.03.597195 | Chioso: Segmentation-free Annotation of Spatial Transcriptomics Data at Sub-cellular Resolution via Adversarial Learning | No | No microscopy imaging |
| 244 | 2024 | bioRxiv | https://doi.org/ 10.1101/2024.06.09.24308649 | Cross-Modality Synthetic Data Augmentation using GANs: Enhancing Brain MRI and Chest X-ray Classification | No | No microscopy imaging |
| 245 | 2024 | bioRxiv | https://doi.org/ 10.1101/2024.06.18.24309092 | Evaluating the Performance of ChatGPT-4o Vision Capabilities on Image-Based USMLE Step 1, Step 2, and Step 3 Examination Questions | No | No microscopy imaging |
| 246 | 2023 | bioRxiv | https://doi.org/ 10.1101/2023.07.11.548246 | RNAGEN: A generative adversarial network-based model to generate synthetic RNA sequences to target proteins | No | No microscopy imaging |
| 247 | 2023 | bioRxiv | https://doi.org/ 10.1101/2023.01.30.526198 | UTRGAN: Learning to Generate 5' UTR Sequences for Optimized Translation Efficiency and Gene Expression | No | No microscopy imaging |
| 248 | 2024 | bioRxiv | https://doi.org/ 10.1101/2024.07.15.603649 | SmartImpute: A Targeted Imputation Framework for Single-cell Transcriptome Data | No | No microscopy imaging |
| 249 | 2024 | bioRxiv | https://doi.org/ 10.1101/2024.07.19.24310732 | Evaluating the Potential of Wearable Technology in Early Stress Detection: A Multimodal Approach | No | No microscopy imaging |
| 250 | 2024 | bioRxiv | https://doi.org/ 10.1101/2024.07.28.24311114 | Comparative Evaluation Of Machine Learning Classifiers For Brain Tumor Detection | No | No microscopy imaging |
| 251 | 2023 | bioRxiv | https://doi.org/ 10.1101/2023.09.26.23296163 | Generative AI Mitigates Representation Bias and Improves Model Fairness Through Synthetic Health Data | No | No microscopy imaging |
| 252 | 2024 | bioRxiv | https://doi.org/ 10.1101/2024.08.21.24312353 | Evaluating Text-to-Image Generated Photorealistic Images of Human Anatomy | No | No microscopy imaging |

| | | | | | | |
|---|---|---|---|---|---|---|
| 253 | 2024 | bioRxiv | https://doi.org/10.1101/2024.09.04.24313070 | Dynamic Contrast Enhanced MRI Mapping of Vascular Permeability for Evaluation of Breast Cancer Neoadjuvant Chemotherapy Response Using Image-to-Image Conditional Generative Adversarial Networks | No | No microscopy imaging |
| 254 | 2024 | bioRxiv | https://doi.org/10.1101/2024.03.25.24304868 | A Novel Digital Twin Strategy to Examine the Implications of Randomized Clinical Trials for Real-World Populations | No | No microscopy imaging |
| 255 | 2024 | bioRxiv | https://doi.org/10.1101/2024.09.03.610823 | Generating virtual patient data for in silico clinical trials of medical devices during extracorporeal membrane oxygenation treatment | No | No microscopy imaging |
| 256 | 2024 | bioRxiv | https://doi.org/10.1101/2024.09.13.24313606 | Generative AI and Large Language Models in Reducing Medication Related Harm and Adverse Drug Events – A Scoping Review | No | No microscopy imaging |
| 257 | 2024 | bioRxiv | https://doi.org/10.1101/2024.10.16.24315609 | Cross-modality image translation of 3 Tesla Magnetic Resonance Imaging to 7 Tesla using Generative Adversarial Networks | No | No microscopy imaging |
| 258 | 2024 | bioRxiv | https://doi.org/10.1101/2024.10.17.618896 | In silico generation of synthetic cancer genomes using generative AI | No | No microscopy imaging |
| 259 | 2024 | bioRxiv | https://doi.org/10.1101/2024.08.07.607012 | Latent generative modeling of long genetic sequences with GANs | No | No microscopy imaging |
| 260 | 2024 | bioRxiv | https://doi.org/10.1101/2024.10.15.24315508 | Enhancing Radiographic Diagnosis: CycleGAN-based methods for reducing cast shadow artifacts in wrist radiographs | No | No microscopy imaging |
| 261 | 2024 | bioRxiv | https://doi.org/10.1101/2024.10.25.620201 | µPIX: Leveraging Generative AI for Enhanced, Personalized and Sustainable Microscopy | No | No image augmentation |
| 262 | 2024 | bioRxiv | https://doi.org/10.1101/2024.04.02.587739 | Bridging Organ Transcriptomics for Advancing Multiple Organ Toxicity Assessment with a Generative AI Approach | No | No microscopy imaging |
| 263 | 2024 | bioRxiv | https://doi.org/10.1101/2024.10.29.620982 | Deep generative design of neutralizing nanobodies against SARS-CoV-2 variants | No | No microscopy imaging |
| 264 | 2023 | bioRxiv | https://doi.org/10.1101/2023.12.19.572452 | ElectroPhysiomeGAN: Generation of Biophysical Neuron Model Parameters from Recorded Electrophysiological Responses | No | No microscopy imaging |
| 265 | 2024 | bioRxiv | https://doi.org/10.1101/2024.11.13.623386 | A Multi-Property Optimizing Generative Adversarial Network for de novo Antimicrobial Peptide Design | No | No microscopy imaging |

| | | | | | | |
|---|---|---|---|---|---|---|
| 266 | 2024 | bioRxiv | https://doi.org/10.1101/2024.01.26.24301803 | HistoPlexer: Histopathology-based Protein Multiplex Generation using Deep Learning | No | No image augmentation |
| 267 | 2024 | bioRxiv | https://doi.org/10.1101/2024.12.16.628596 | Natural speech re-synthesis from direct cortical recordings using a pre-trained encoder-decoder framework | No | No microscopy imaging |
| 268 | 2025 | bioRxiv | https://doi.org/10.1101/2025.01.26.25321159 | Schizophrenia versus Healthy Controls Classification based on fMRI 4D Spatiotemporal Data | No | No microscopy imaging |
| 269 | 2025 | bioRxiv | https://doi.org/10.1101/2025.02.06.636827 | WormAI: Artificial Intelligence Networks for Nematode Phenotyping | No | No image augmentation |
| 270 | 2025 | bioRxiv | https://doi.org/10.1101/2025.02.10.25321126 | Leveraging Deep Learning to Enhance MRI for Brain Disorders | No | No microscopy imaging |
| 271 | 2025 | bioRxiv | https://doi.org/10.1101/2025.02.12.637853 | Accurate de novo transcription unit annotation from run-on and sequencing data | No | No microscopy imaging |
| 272 | 2025 | bioRxiv | https://doi.org/10.1101/2025.03.04.641434 | Intelligent Design of Escherichia coli Terminators | No | No microscopy imaging |
| 273 | 2025 | bioRxiv | https://doi.org/10.1101/2025.03.07.25322919 | Deep generative models for vessel segmentation in CT angiography of the brain | No | No microscopy imaging |
| 274 | 2025 | bioRxiv | https://doi.org/10.1101/2025.04.07.646944 | Harmonizing Inter-Site Differences in T1-Weighted Images Using CycleGAN | No | No microscopy imaging |
| 275 | 2020 | arXiv | https://doi.org/10.48550/arXiv.2002.00647 | Pix2Pix-based Stain-to-Stain Translation: A Solution for Robust Stain Normalization in Histopathology Images Analysis | No | No image augmentation |
| 276 | 2020 | arXiv | https://doi.org/10.48550/arXiv.2004.06517 | Learning a low dimensional manifold of real cancer tissue with PathologyGAN | **Yes** | N/A |
| 277 | 2021 | arXiv | https://doi.org/10.48550/arXiv.2104.09435 | Deep learning enables reference-free isotropic super-resolution for volumetric fluorescence microscopy | No | No image augmentation |
| 278 | 2020 | arXiv | https://doi.org/10.48550/arXiv.2003.13653 | Vox2Vox: 3D-GAN for Brain Tumour Segmentation | No | No microscopy imaging |
| 279 | 2020 | arXiv | https://doi.org/10.48550/arXiv.2009.09574 | Reconstruct high-resolution multi-focal plane images from a single 2D wide field image | No | No image augmentation |
| 280 | 2019 | arXiv | https://doi.org/10.48550/arXiv.1901.06219 | Red blood cell image generation for data augmentation using Conditional Generative Adversarial Networks | No | No image augmentation |

| | | | | | | |
|---|---|---|---|---|---|---|
| 281 | 2019 | arXiv | https://doi.org/10.48550/ arXiv.1905.11034 | Unsupervised Learning of Anomaly Detection from Contaminated Image Data using Simultaneous Encoder Training | No | No image augmentation |
| 282 | 2018 | arXiv | https://doi.org/10.48550/ arXiv.1810.00236 | Deep Adversarial Training for Multi-Organ Nuclei Segmentation in Histopathology Images | No | No image augmentation |
| 283 | 2022 | arXiv | https://doi.org/10.48550/ arXiv.2207.14650 | SYNTA: A novel approach for deep learning-based image analysis in muscle histopathology using photo-realistic synthetic data | No | No image augmentation |
| 284 | 2020 | arXiv | https://doi.org/10.48550/ arXiv.2007.12098 | Optimal Transport using GANs for Lineage Tracing | No | No microscopy imaging |
| 285 | 2022 | arXiv | https://doi.org/10.1109/ CIBCB56990.2023.10264899 | A SSIM Guided cGAN Architecture For Clinically Driven Generative Image Synthesis of Multiplexed Spatial Proteomics Channels | No | No image augmentation |
| 286 | 2017 | arXiv | https://doi.org/10.48550/ arXiv.1708.04692 | GANs for Biological Image Synthesis | **Yes** | N/A |
| 287 | 2017 | arXiv | https://doi.org/10.48550/ arXiv.1711.11317 | Unsupervised Learning for Cell-level Visual Representation in Histopathology Images with Generative Adversarial Networks | **Yes** | N/A |
| 288 | 2018 | arXiv | https://doi.org/10.48550/ arXiv.1811.04498 | Product Title Refinement via Multi-Modal Generative Adversarial Learning | No | No microscopy imaging |
| 289 | 2022 | arXiv | https://doi.org/10.48550/ arXiv.2201.04302 | De-Noising of Photoacoustic Microscopy Images by Deep Learning | No | No microscopy imaging |
| 290 | 2018 | arXiv | https://doi.org/10.48550/ arXiv.1907.06143 | Neural Embedding for Physical Manipulations | No | No microscopy imaging |
| 291 | 2021 | arXiv | https://doi.org/10.48550/ arXiv.2101.03549 | Learning Rotation Invariant Features for Cryogenic Electron Microscopy Image Reconstruction | No | No image augmentation |
| 292 | 2018 | arXiv | https://doi.org/10.48550/ arXiv.1804.00393 | Generative Spatiotemporal Modeling Of Neutrophil Behavior | No | No image augmentation |
| 293 | 2018 | arXiv | https://doi.org/10.48550/ arXiv.1805.04634 | Image-derived generative modeling of pseudo-macromolecular structures - towards the statistical assessment of Electron CryoTomography template matching | No | No image augmentation |
| 294 | 2020 | arXiv | https://doi.org/10.48550/ arXiv.2012.12561 | GANDA: A deep generative adversarial network predicts the spatial distribution of nanoparticles in tumor pixelly | No | No image augmentation |

| | | | | | | |
|---|---|---|---|---|---|---|
| 295 | 2021 | arXiv | https://doi.org/10.48550/ arXiv.2103.11834 | Generation and Simulation of Yeast Microscopy Imagery with Deep Learning | No | No image augmentation |
| 296 | 2021 | arXiv | https://doi.org/10.48550/ arXiv.1911.08121 | Estimation of Orientation and Camera Parameters from Cryo-Electron Microscopy Images with Variational Autoencoders and Generative Adversarial Networks | No | No image augmentation |
| 297 | 2018 | arXiv | https://doi.org/10.48550/ arXiv.1801.07330 | High-throughput, high-resolution registration-free generated adversarial network microscopy | No | No image augmentation |
| 298 | 2022 | arXiv | https://doi.org/10.48550/ arXiv.2202.01115 | NeuRegenerate: A Framework for Visualizing Neurodegeneration | No | No image augmentation |
| 299 | 2021 | arXiv | https://doi.org/10.48550/ arXiv.2103.02588 | IH-GAN: A Conditional Generative Model for Implicit Surface-Based Inverse Design of Cellular Structures | No | No image augmentation |
| 300 | 2019 | arXiv | https://doi.org/10.48550/ arXiv.1904.01947 | Extracting Tables from Documents using Conditional Generative Adversarial Networks and Genetic Algorithms | No | No microscopy imaging |
| 301 | 2018 | arXiv | https://doi.org/10.48550/ arXiv.1803.00385 | MAGAN: Aligning Biological Manifolds | No | No microscopy imaging |
| 302 | 2022 | arXiv | https://doi.org/10.48550/ arXiv.2209.11923 | DeepChrome 2.0: Investigating and Improving Architectures, Visualizations, & Experiments | No | No microscopy imaging |
| 303 | 2021 | arXiv | https://doi.org/10.48550/ arXiv.2108.04328 | Generative Adversarial Neural Cellular Automata | No | No microscopy imaging |
| 304 | 2020 | arXiv | https://doi.org/10.48550/ arXiv.2010.15315 | Exploring Generative Adversarial Networks for Image-to-Image Translation in STEM Simulation | No | No microscopy imaging |
| 305 | 2018 | arXiv | https://doi.org/10.48550/ arXiv.1811.03815 | Neural Stain Normalization and Unsupervised Classification of Cell Nuclei in Histopathological Breast Cancer Images | No | No image augmentation |
| 306 | 2022 | arXiv | https://doi.org/10.48550/ arXiv.2206.08308 | Deepfake histological images for enhancing digital pathology | No | No image augmentation |
| 307 | 2020 | arXiv | https://doi.org/10.48550/ arXiv.2011.07466 | Continuous Conditional Generative Adversarial Networks: Novel Empirical Losses and Label Input Mechanisms | No | No microscopy imaging |
| 308 | 2018 | arXiv | https://doi.org/10.48550/ arXiv.1803.11293 | Deep learning-based virtual histology staining using auto-fluorescence of label-free tissue | No | No image augmentation |

| | | | | | | |
|---|---|---|---|---|---|---|
| 309 | 2022 | arXiv | https://doi.org/10.48550/arXiv.2208.08284 | Novel Deep Learning Approach to Derive Cytokeratin Expression and Epithelium Segmentation from DAPI | No | No image augmentation |
| 310 | 2022 | arXiv | https://doi.org/10.48550/arXiv.2204.03082 | Instance Segmentation of Unlabeled Modalities via Cyclic Segmentation GAN | No | No image augmentation |
| 311 | 2020 | arXiv | https://doi.org/10.48550/arXiv.2003.11632 | Pores for thought: The use of generative adversarial networks for the stochastic reconstruction of 3D multi-phase electrode microstructures with periodic boundaries | No | No microscopy imaging |
| 312 | 2019 | arXiv | https://doi.org/10.48550/arXiv.1909.00240 | Integrating Data and Image Domain Deep Learning for Limited Angle Tomography using Consensus Equilibrium | No | No microscopy imaging |
| 313 | 2021 | arXiv | https://doi.org/10.48550/arXiv.2111.12138 | Multi-Modality Microscopy Image Style Transfer for Nuclei Segmentation | No | No image augmentation |
| 314 | 2019 | arXiv | https://doi.org/10.48550/arXiv.1909.12116 | Optimal Transport driven CycleGAN for Unsupervised Learning in Inverse Problems | No | No microscopy imaging |
| 315 | 2019 | arXiv | https://doi.org/10.48550/arXiv.1901.11094 | Resolution enhancement in scanning electron microscopy using deep learning | No | No image augmentation |
| 316 | 2022 | arXiv | https://doi.org/10.48550/arXiv.2207.10541 | Optimal precision for GANs | No | No microscopy imaging |
| 317 | 2021 | arXiv | https://doi.org/10.48550/arXiv.2104.04953 | SIGAN: A Novel Image Generation Method for Solar Cell Defect Segmentation and Augmentation | No | No image augmentation |
| 318 | 2020 | arXiv | https://doi.org/10.48550/arXiv.2008.01124 | Analyzing the Components of Distributed Coevolutionary GAN Training | No | No microscopy imaging |
| 319 | 2021 | arXiv | https://doi.org/10.48550/arXiv.2106.06920 | Multi-modal Scene-compliant User Intention Estimation in Navigation | No | No microscopy imaging |
| 320 | 2020 | arXiv | https://doi.org/10.48550/arXiv.2009.08267 | Integration of AI and mechanistic modeling in generative adversarial networks for stochastic inverse problems | No | No microscopy imaging |
| 321 | 2019 | arXiv | https://doi.org/10.48550/arXiv.1906.00078 | Augmenting C. elegans Microscopic Dataset for Accelerated Pattern Recognition | **Yes** | N/A |
| 322 | 2022 | arXiv | https://doi.org/10.48550/arXiv.2203.08289 | Driving Anomaly Detection Using Conditional Generative Adversarial Network | No | No microscopy imaging |

| | | | | | | |
|---|---|---|---|---|---|---|
| 323 | 2021 | arXiv | https://doi.org/10.48550/arXiv.2103.03518 | Anomaly detection and automatic labeling for solar cell quality inspection based on Generative Adversarial Network | No | No microscopy imaging |
| 324 | 2021 | arXiv | https://doi.org/10.48550/arXiv.2107.04036 | Pattern Detection on Glioblastoma's Waddington landscape via Generative Adversarial Networks | No | No microscopy imaging |
| 325 | 2021 | arXiv | https://doi.org/10.48550/arXiv.2109.09004 | Random Multi-Channel Image Synthesis for Multiplexed Immunofluorescence Imaging | No | No image augmentation |
| 326 | 2021 | arXiv | https://doi.org/10.48550/arXiv.2101.01178 | Advances in Electron Microscopy with Deep Learning | No | No image augmentation |
| 327 | 2017 | arXiv | https://doi.org/10.48550/arXiv.1707.04582 | Capturing the diversity of biological tuning curves using generative adversarial networks | No | No microscopy imaging |
| 328 | 2018 | arXiv | https://doi.org/10.48550/arXiv.1807.07701 | PhaseStain: Digital staining of label-free quantitative phase microscopy images using deep learning | No | No image augmentation |
| 329 | 2018 | arXiv | https://doi.org/10.48550/arXiv.1802.09070 | Attention-Aware Generative Adversarial Networks (ATA-GANs) | **Yes** | N/A |
| 330 | 2020 | arXiv | https://doi.org/10.48550/arXiv.2006.13886 | Microstructure Generation via Generative Adversarial Network for Heterogeneous, Topologically Complex 3D Materials | No | No microscopy imaging |
| 331 | 2021 | arXiv | https://doi.org/10.48550/arXiv.2109.09702 | Deep Anomaly Generation: An Image Translation Approach of Synthesizing Abnormal Banded Chromosome Images | No | No image augmentation |
| 332 | 2020 | arXiv | https://doi.org/10.48550/arXiv.2010.04634 | Attaining Real-Time Super-Resolution for Microscopic Images Using GAN | No | No image augmentation |
| 333 | 2022 | arXiv | https://doi.org/10.48550/arXiv.2203.02940 | Detection of Parasitic Eggs from Microscopy Images and the emergence of a new dataset | No | No image augmentation |
| 334 | 2020 | arXiv | https://doi.org/10.48550/arXiv.2011.00189 | Enhanced Balancing GAN: Minority-class Image Generation | No | No microscopy imaging |
| 335 | 2019 | arXiv | https://doi.org/10.48550/arXiv.1907.03728 | Correlation via synthesis: end-to-end nodule image generation and radiogenomic map learning based on generative adversarial network | No | No image augmentation |
| 336 | 2020 | arXiv | https://doi.org/10.48550/arXiv.2004.14936 | Generative Adversarial Networks in Digital Pathology: A Survey on Trends and Future Potential | No | No image augmentation |

| | | | | | | |
|---|---|---|---|---|---|---|
| 337 | 2019 | arXiv | https://doi.org/10.48550/arXiv.1911.04410 | A deep learning framework for morphologic detail beyond the diffraction limit in infrared spectroscopic imaging | No | No image augmentation |
| 338 | 2019 | arXiv | https://doi.org/10.48550/arXiv.1909.04518 | Virtual organelle self-coding for fluorescence imaging via adversarial learning | No | No image augmentation |
| 339 | 2018 | arXiv | https://doi.org/10.48550/arXiv.1806.08666 | Combining Recurrent Neural Networks and Adversarial Training for Human Motion Synthesis and Control | No | No microscopy imaging |
| 340 | 2022 | arXiv | https://doi.org/10.48550/arXiv.2201.03597 | Cross-Modality Sub-Image Retrieval using Contrastive Multimodal Image Representations | No | No image augmentation |
| 341 | 2018 | arXiv | https://doi.org/10.48550/arXiv.1812.11006 | TOP-GAN: Label-Free Cancer Cell Classification Using Deep Learning with a Small Training Set | **Yes** | N/A |
| 342 | 2019 | arXiv | https://doi.org/10.48550/arXiv.1908.09414 | CycleGAN with a Blur Kernel for Deconvolution Microscopy: Optimal Transport Geometry | No | No image augmentation |
| 343 | 2021 | arXiv | https://doi.org/10.48550/arXiv.2107.10180 | 3D fluorescence microscopy data synthesis for segmentation and benchmarking | **Yes** | N/A |
| 344 | 2021 | arXiv | https://doi.org/10.48550/arXiv.2102.08929 | Signal Propagation in a Gradient-Based and Evolutionary Learning System | No | No microscopy imaging |
| 345 | 2019 | arXiv | https://doi.org/10.48550/arXiv.1907.06727 | Deep learning-based color holographic microscopy | No | No image augmentation |
| 346 | 2020 | arXiv | https://doi.org/10.48550/arXiv.2001.05853 | Identifying Table Structure in Documents using Conditional Generative Adversarial Networks | No | No microscopy imaging |
| 347 | 2021 | arXiv | https://doi.org/10.48550/arXiv.2109.08622 | Harnessing Optoelectronic Noises in a Photonic Generative Network | No | No microscopy imaging |
| 348 | 2019 | arXiv | https://doi.org/10.48550/arXiv.1902.08716 | Spatio-Temporal Convolutional LSTMs for Tumor Growth Prediction by Learning 4D Longitudinal Patient Data | No | No microscopy imaging |
| 349 | 2019 | arXiv | https://doi.org/10.48550/arXiv.1910.14207 | Multi-defect microscopy image restoration under limited data conditions | No | No image augmentation |
| 350 | 2022 | arXiv | https://doi.org/10.48550/arXiv.2203.03489 | DATGAN: Integrating expert knowledge into deep learning for synthetic tabular data | No | No microscopy imaging |
| 351 | 2018 | arXiv | https://doi.org/10.48550/arXiv.1805.00334 | Deep learning approach to Fourier ptychographic microscopy | No | No image augmentation |

| | | | | | | |
|---|---|---|---|---|---|---|
| 352 | 2020 | arXiv | https://doi.org/10.48550/arXiv.2004.00140 | Learning Generative Models of Tissue Organization with Supervised GANs | **Yes** | N/A |
| 353 | 2021 | arXiv | https://doi.org/10.48550/arXiv.2106.08285 | Multi-StyleGAN: Towards Image-Based Simulation of Time-Lapse Live-Cell Microscopy | **Yes** | N/A |
| 354 | 2021 | arXiv | https://doi.org/10.48550/arXiv.2109.12404 | Deep learning tackles single-cell analysis A survey of deep learning for scRNA-seq analysis | No | No microscopy imaging |
| 355 | 2018 | arXiv | https://doi.org/10.48550/arXiv.1811.02642 | Computational Histological Staining and Destaining of Prostate Core Biopsy RGB Images with Generative Adversarial Neural Networks | No | No image augmentation |
| 356 | 2018 | arXiv | https://doi.org/10.48550/arXiv.1804.03700 | Unsupervised and semi-supervised learning with Categorical Generative Adversarial Networks assisted by Wasserstein distance for dermoscopy image Classification | No | No microscopy imaging |
| 357 | 2020 | arXiv | https://doi.org/10.48550/arXiv.2007.12578 | Stain Style Transfer of Histopathology Images Via Structure-Preserved Generative Learning | No | No image augmentation |
| 358 | 2019 | arXiv | https://doi.org/10.48550/arXiv.1910.06428 | Restoration of marker occluded hematoxylin and eosin stained whole slide histology images using generative adversarial networks | No | No image augmentation |
| 359 | 2021 | arXiv | https://doi.org/10.48550/arXiv.2107.11022 | AD-GAN: End-to-end Unsupervised Nuclei Segmentation with Aligned Disentangling Training | No | No image augmentation |
| 360 | 2021 | arXiv | https://doi.org/10.48550/arXiv.2107.07761 | Exploiting generative self-supervised learning for the assessment of biological images with lack of annotations: a COVID-19 case-study | **Yes** | N/A |
| 361 | 2021 | arXiv | https://doi.org/10.48550/arXiv.2107.12357 | Structure-Preserving Multi-Domain Stain Color Augmentation using Style-Transfer with Disentangled Representations | No | No image augmentation |
| 362 | 2019 | arXiv | https://doi.org/10.48550/arXiv.1908.03841 | Transcriptional Response of SK-N-AS Cells to Methamidophos | No | No microscopy imaging |
| 363 | 2022 | arXiv | https://doi.org/10.48550/arXiv.2209.13283 | A comparative study of attention mechanism and generative adversarial network in facade damage segmentation | No | No microscopy imaging |
| 364 | 2022 | arXiv | https://doi.org/10.48550/arXiv.2210.06909 | HoechstGAN: Virtual Lymphocyte Staining Using Generative Adversarial Networks | No | No image augmentation |
| 365 | 2022 | arXiv | https://doi.org/10.48550/arXiv.2211.00829 | Exploiting Spatial-temporal Correlations for Video Anomaly Detection | No | No microscopy imaging |

| | | | | | | |
|---|---|---|---|---|---|---|
| 366 | 2022 | arXiv | https://doi.org/10.48550/arXiv.2211.06150 | Improved HER2 Tumor Segmentation with Subtype Balancing using Deep Generative Networks | No | No image augmentation |
| 367 | 2022 | arXiv | https://doi.org/10.48550/arXiv.2211.06146 | An unobtrusive quality supervision approach for medical image annotation | **Yes** | N/A |
| 368 | 2022 | arXiv | https://doi.org/10.48550/arXiv.2211.06522 | Deep Learning Generates Synthetic Cancer Histology for Explainability and Education | **Yes** | |
| 369 | 2022 | arXiv | https://doi.org/10.48550/arXiv.2212.10478 | Machine Learning and Polymer Self-Consistent Field Theory in Two Spatial Dimensions | No | No microscopy imaging |
| 370 | 2022 | arXiv | https://doi.org/10.48550/arXiv.2301.01253 | Deep Learning for bias-correcting comprehensive high-resolution Earth system models | No | No microscopy imaging |
| 371 | 2022 | arXiv | https://doi.org/10.48550/arXiv.2301.01253 | Using CycleGANs to Generate Realistic STEM Images for Machine Learning | No | No microscopy imaging |
| 372 | 2022 | arXiv | https://doi.org/10.48550/arXiv.2211.14753 | A Self-adaptive Neuroevolution Approach to Constructing Deep Neural Network Architectures Across Different Types | No | No microscopy imaging |
| 373 | 2023 | arXiv | https://doi.org/10.48550/arXiv.2302.01104 | LesionAid: Vision Transformers-based Skin Lesion Generation and Classification | No | No microscopy imaging |
| 374 | 2023 | arXiv | https://doi.org/10.48550/arXiv.2302.03120 | Studying Therapy Effects and Disease Outcomes in Silico using Artificial Counterfactual Tissue Samples | No | No microscopy imaging |
| 375 | 2023 | arXiv | https://doi.org/10.48550/arXiv.2302.06549 | Between Generating Noise and Generating Images: Noise in the Correct Frequency Improves the Quality of Synthetic Histopathology Images for Digital Pathology | No | No image augmentation |
| 376 | 2023 | arXiv | https://doi.org/10.48550/arXiv.2303.02057 | Unsupervised Deep Digital Staining For Microscopic Cell Images Via Knowledge Distillation | No | No image augmentation |
| 377 | 2023 | arXiv | https://doi.org/10.48550/arXiv.2301.07743 | Leveraging generative adversarial networks to create realistic scanning transmission electron microscopy images | No | No microscopy imaging |
| 378 | 2023 | arXiv | https://doi.org/10.48550/arXiv.2303.15214 | Generalizable Denoising of Microscopy Images using Generative Adversarial Networks and Contrastive Learning | No | No image augmentation |
| 379 | 2023 | arXiv | https://doi.org/10.48550/arXiv.2304.10065 | Machine learning traction force maps of cell monolayers | No | No image augmentation |

| | | | | | | |
|---|---|---|---|---|---|---|
| 380 | 2023 | arXiv | https://doi.org/10.48550/arXiv.2306.00548 | Label- and slide-free tissue histology using 3D epi-mode quantitative phase imaging and virtual H&E staining | No | No image augmentation |
| 381 | 2023 | arXiv | https://doi.org/10.48550/arXiv.2307.12138 | SCPAT-GAN: Structural Constrained and Pathology Aware Convolutional Transformer-GAN for Virtual Histology Staining of Human Coronary OCT images | No | No image augmentation |
| 382 | 2023 | arXiv | https://doi.org/10.48550/arXiv.2307.14262 | Artifact Restoration in Histology Images with Diffusion Probabilistic Models | No | No image augmentation |
| 383 | 2023 | arXiv | https://doi.org/10.48550/arXiv.2310.10414 | Style transfer between Microscopy and Magnetic Resonance Imaging via Generative Adversarial Network in small sample size settings | No | No image augmentation |
| 384 | 2023 | arXiv | https://doi.org/10.48550/arXiv.2311.10305 | Semi-supervised ViT knowledge distillation network with style transfer normalization for colorectal liver metastases survival prediction | No | No image augmentation |
| 385 | 2024 | arXiv | https://doi.org/10.48550/arXiv.2405.04211 | Leveraging Medical Foundation Model Features in Graph Neural Network-Based Retrieval of Breast Histopathology Images | No | No image augmentation |
| 386 | 2024 | arXiv | https://doi.org/10.48550/arXiv.2405.13278 | Single color digital H&E staining with In-and-Out Net | No | No image augmentation |
| 387 | 2024 | arXiv | https://doi.org/10.48550/arXiv.2407.20172 | LatentArtiFusion: An Effective and Efficient Histological Artifacts Restoration Framework | No | No image augmentation |
| 388 | 2024 | arXiv | https://doi.org/10.48550/arXiv.2408.15218 | Histo-Diffusion: A Diffusion Super-Resolution Method for Digital Pathology with Comprehensive Quality Assessment | No | No image augmentation |
| 389 | 2024 | arXiv | https://doi.org/10.48550/arXiv.2409.08338 | Impact of Stain Variation and Color Normalization for Prognostic Predictions in Pathology | No | No image augmentation |
| 390 | 2025 | arXiv | https://doi.org/10.48550/arXiv.2501.03592 | A Value Mapping Virtual Staining Framework for Large-scale Histological Imaging | No | No image augmentation |
| 391 | 2025 | arXiv | https://doi.org/10.48550/arXiv.2504.04130 | Scaling Federated Learning Solutions with Kubernetes for Synthesizing Histopathology Images | **Yes** | N/A |
| 392 | 2021 | IEEE | 10.1109/CASE49439.2021.9551632 | Automated Domain Adaptation in Tool Condition Monitoring using Generative Adversarial Networks | No | No microscopy imaging |

| | | | | | | |
|---|---|---|---|---|---|---|
| 393 | 2020 | IEEE | 10.1109/ CVPRW50498.2020.00493 | Estimation of Orientation and Camera Parameters from Cryo-Electron Microscopy Images with Variational Autoencoders and Generative Adversarial Networks | No | No image augmentation |
| 394 | 2021 | IEEE | 10.1109/ ISBI48211.2021.9433955 | Automated Segmentation Of Corneal Nerves In Confocal Microscopy Via Contrastive Learning Based Synthesis And Quality Enhancement | No | No image augmentation |
| 395 | 2021 | IEEE | 10.1109/TVCG.2019.2938520 | Combining Recurrent Neural Networks and Adversarial Training for Human Motion Synthesis and Control | No | No microscopy imaging |
| 396 | 2021 | IEEE | 10.1109/ ACCESS.2021.3084597 | TilGAN: GAN for Facilitating Tumor-Infiltrating Lymphocyte Pathology Image Synthesis With Improved Image Classification | No | No image augmentation |
| 397 | 2019 | IEEE | 10.1109/CVPRW.2019.00136 | Red Blood Cell Image Generation for Data Augmentation Using Conditional Generative Adversarial Networks | No | No image augmentation |
| 398 | 2022 | IEEE | 10.1109/TII.2022.3168667 | Small sample reliability assessment with online time-series data based on a worm WGAN learning method | No | No microscopy imaging |
| 399 | 2021 | IEEE | 10.1109/ PerComWorkshops51409.2021. 9431046 | Data Augmentation Strategies for Human Activity Data Using Generative Adversarial Neural Networks | No | No microscopy imaging |
| 400 | 2021 | IEEE | 10.1109/ ACCESS.2021.3088893 | ZEM: Zero-Cycle Bit-Masking Module for Deep Learning Refresh-Less DRAM | No | No microscopy imaging |
| 401 | 2022 | IEEE | 10.1109/TVT.2022.3157802 | A Data-Driven Framework for Inter-Frequency Handover Failure Prediction and Mitigation | No | No microscopy imaging |
| 402 | 2020 | IEEE | 10.1109/CVPRW.2019.00145 | Cell Image Segmentation Using Generative Adversarial Networks, Transfer Learning, and Augmentations | No | No image augmentation |
| 403 | 2021 | IEEE | 10.1109/ ESCI50559.2021.9396991 | Automating Generative Adversarial Networks using Neural Architecture Search: A Review | No | No microscopy imaging |
| 404 | 2018 | IEEE | 10.1109/WACV.2018.00080 | Learning Generative Models of Tissue Organization with Supervised GANs | **Yes** | N/A |
| 405 | 2020 | IEEE | 10.1109/ ISBI45749.2020.9098618 | ISING-GAN: Annotated Data Augmentation with a Spatially Constrained Generative Adversarial Network | **Yes** | N/A |
| 406 | 2022 | IEEE | 10.1109/ | Attention Residual Network for White Blood Cell Classification with WGAN Data | **Yes** | N/A |

| | | | | | | |
|---|---|---|---|---|---|---|
| | | | ITME53901.2021.00075 | Augmentation | | |
| 407 | 2021 | IEEE | 10.1109/ BHI50953.2021.9508532 | Improving Heart Transplant Rejection Classification Training using Progressive Generative Adversarial Networks | **Yes** | N/A |
| 408 | 2017 | IEEE | 10.1109/ICCV.2017.245 | GANs for Biological Image Synthesis | **Yes** | N/A |
| 409 | 2020 | IEEE | 10.1109/ ICAIBD49809.2020.9137494 | Cervical Cancer Single Cell Image Data Augmentation Using Residual Condition Generative Adversarial Networks | **Yes** | N/A |
| 410 | 2022 | IEEE | 10.1109/ ACCESS.2022.3178786 | Deep Anomaly Generation: An Image Translation Approach of Synthesizing Abnormal Banded Chromosome Images | No | No image augmentation |
| 411 | 2019 | IEEE | 10.1109/ISBI.2019.8759199 | Towards the Identification of Histology Based Subtypes in Prostate Cancer | No | No image augmentation |
| 412 | 2022 | IEEE | 10.1109/ ICAIIC54071.2022.9722696 | Addressing Data Sparsity with GANs for Multi-fault Diagnosing in Emerging Cellular Networks | No | No microscopy imaging |
| 413 | 2022 | IEEE | 10.23919/JCC.2022.08.013 | An efficient correlation-aware anomaly detection framework in cellular network | No | No microscopy imaging |
| 414 | 2019 | IEEE | 10.1109/ICIP.2019.8802971 | Quality Assessment of Synthetic Fluorescence Microscopy Images for Image Segmentation | No | No image augmentation |
| 415 | 2021 | IEEE | 10.1109/ ACCESS.2021.3095391 | Studying the Applicability of Generative Adversarial Networks on HEp-2 Cell Image Augmentation | **Yes** | N/A |
| 416 | 2021 | IEEE | 10.1109/ IPFA53173.2021.9617416 | Generative Adversarial Network for Integrated Circuits Physical Assurance Using Scanning Electron Microscopy | No | No microscopy imaging |
| 417 | 2022 | IEEE | 10.1109/TAP.2021.3138517 | Prior-Knowledge-Guided Deep-Learning-Enabled Synthesis for Broadband and Large Phase Shift Range Metacells in Metalens Antenna | No | No microscopy imaging |
| 418 | 2020 | IEEE | 10.1109/ TCSET49122.2020.235565 | Deep Learning based Mobile Network Management for 5G and Beyond | No | No microscopy imaging |
| 419 | 2022 | IEEE | 10.1109/JIOT.2022.3152729 | GAN for Load Estimation and Traffic-Aware Network Selection for 5G Terminals | No | No microscopy imaging |
| 420 | 2021 | IEEE | 10.1109/ ACCESS.2021.3104609 | Segmentation of Cervical Cell Images Based on Generative Adversarial Networks | No | No image augmentation |

| | | | | | | |
|---|---|---|---|---|---|---|
| 421 | 2021 | IEEE | 10.1109/ ICASSP39728.2021.9413892 | Improving Stability of Adversarial Li-ion Cell Usage Data Generation using Generative Latent Space Modelling | No | No microscopy imaging |
| 422 | 2022 | IEEE | 10.1109/ IJCNN55064.2022.9892954 | MID3A: Microscopy Image Denoising meets Differentiable Data Augmentation | No | No image augmentation |
| 423 | 2022 | IEEE | 10.1109/ SMC53654.2022.9945563 | Dual Generative Adversarial Network For Ultrasound Localization Microscopy | No | No microscopy imaging |
| 424 | 2022 | IEEE | 10.1109/ COMNETSAT56033.2022.9994559 | DCGAN-based Medical Image Augmentation to Improve ELM Classification Performance | **Yes** | N/A |
| 425 | 2022 | IEEE | 10.1109/ ICAISS55157.2022.10010838 | White Blood Cell Image Generation using Deep Convolutional Generative Adversarial Network | **Yes** | N/A |
| 426 | 2022 | IEEE | 10.1109/ IECBES54088.2022.10079623 | A Hybrid Ensemble Learning with Generative Adversarial Networks for HEp-2 Cell Image Classification | **Yes** | N/A |
| 427 | 2022 | IEEE | 10.1109/ ICCSMT58129.2022.00045 | GOSGAN: An Adaptive Atomic Force Microscope Imaging Repair Model for Cell Scanning | No | No image augmentation |
| 428 | 2023 | IEEE | 10.1109/ IC3S57698.2023.10169363 | A Hybrid Approach for Improving the Classification performance of Imbalanced Breast Cancer data | **Yes** | N/A |
| 429 | 2023 | IEEE | 10.1109/ ICTON59386.2023.10207523 | Super-Resolved Non-linear Optical Microscopy: Architectures, Advantages and Perspectives | No | No image augmentation |
| 430 | 2023 | IEEE | 10.1109/ ICIP49359.2023.10222546 | Style Transfer Between Microscopy and Magnetic Resonance Imaging Via Generative Adversarial Network in Small Sample Size Settings | No | No image augmentation |
| 431 | 2023 | IEEE | 10.1109/TMI.2023.3314695 | Digital Staining of White Blood Cells With Confidence Estimation | No | No image augmentation |
| 432 | 2023 | IEEE | 10.23919/ CCC58697.2023.10240781 | Generation of Solar Cell Defect Images Based on Multi-Perceptual Fields and Attention Mechanism | No | No microscopy imaging |
| 433 | 2023 | IEEE | 10.1109/ DSAA60987.2023.10302513 | Sparse Self-Attention Guided Generative Adversarial Networks for Time-Series Generation | No | No microscopy imaging |
| 434 | 2023 | IEEE | 10.1109/ BHI58575.2023.10313485 | RNA sequencing-based histological subtyping of non-small cell lung cancer with generative adversarial data imputation | No | No microscopy imaging |

| | | | | | | |
|---|---|---|---|---|---|---|
| 435 | 2023 | IEEE | 10.1109/ ACCESS.2023.3332628 | The Power of Generative AI to Augment for Enhanced Skin Cancer Classification: A Deep Learning Approach | No | No microscopy imaging |
| 436 | 2023 | IEEE | 10.1109/ BigData59044.2023.10386596 | Mask-cscGAN for realistic synthetic cell generation | No | No microscopy imaging |
| 437 | 2023 | IEEE | 10.1109/TSM.2023.3327784 | Hotspot Prediction: SEM Image Generation With Potential Lithography Hotspots | No | No microscopy imaging |
| 438 | 2024 | IEEE | 10.1109/ ISIVC61350.2024.10577933 | Generating 3D Microstructure Images for O2 Fuel Cell Electrode using GANs Enhanced with Minkowski Functionals | No | No microscopy imaging |
| 439 | 2024 | IEEE | 10.1109/ITC-CSCC62988.2024.10628337 | A Generative Adversarial Network-Based Approach for Reflective-Metasurface Unit-Cell Synthesis in mmWave Bands | No | No microscopy imaging |
| 440 | 2024 | IEEE | 10.1109/ ICESC60852.2024.10689851 | Deep Neural Networks for Dermatology: CNN-GAN in Multi-Class Skin Disease Detection | No | No microscopy imaging |
| 441 | 2024 | IEEE | 10.1109/IIST62526.2024.00140 | Research on the Application of Deep Learning in Biomedical Image Processing | No | No microscopy imaging |
| 442 | 2024 | IEEE | 10.1109/ ASYU62119.2024.10757024 | Generating Robust Adversarial Images in Medical Image Classification using GANs | No | No image augmentation |
| 443 | 2024 | IEEE | 10.1109/UFFC-JS60046.2024.10793898 | Direct Diffusion Bridge for High MB-count Image Generation in Ultrasound Localization Microscopy | No | No microscopy imaging |
| 444 | 2024 | IEEE | 10.1109/ BIBE63649.2024.10820474 | Depression Diagnosis: Bioinformatics Uncovers Cellular Abnormalities in Female-Specific Genes With Ai-Driven Blood Biomarker-Based Prediction | No | No microscopy imaging |
| 445 | 2024 | IEEE | 10.1109/ BigData62323.2024.10825005 | Melanoma Classification using GAN based augmentation and Self-Supervised feature extraction | No | No microscopy imaging |
| 446 | 2025 | IEEE | 10.23919/USNC-URSINRSM66067.2025.10906961 | Reconfigurable Transmitarray Design with Generative Adversarial Network | No | No microscopy imaging |
| 447 | 2020 | PubMed | 10.1117/12.2549891 | Generative modeling for renal microanatomy | **Yes** | N/A |
| 448 | 2021 | PubMed | 10.1093/bib/bbab160 | DeepImmuno: deep learning-empowered prediction and generation of immunogenic peptides for T-cell immunity | No | No microscopy imaging |

| | | | | | | |
|---|---|---|---|---|---|---|
| 449 | 2021 | PubMed | 10.1016/j.heliyon.2021.e06331 | Mutual stain conversion between Giemsa and Papanicolaou in cytological images using cycle generative adversarial network | No | No image augmentation |
| 450 | 2021 | PubMed | PMC8730359 | Random Multi-Channel Image Synthesis for Multiplexed Immunofluorescence Imaging | No | No image augmentation |
| 451 | 2020 | PubMed | 10.1371/journal.pone.0229951 | Deep learning approach to classification of lung cytological images: Two-step training using actual and synthesized images by progressive growing of generative adversarial networks | **Yes** | N/A |
| 452 | 2022 | PubMed | 10.1117/12.2611827 | Inpainting Missing Tissue in Multiplexed Immunofluorescence Imaging | No | Not available |
| 453 | 2022 | PubMed | 10.1016/j.cmpb.2021.106578 | Super-resolution reconstruction of pneumocystis carinii pneumonia images based on generative confrontation network | No | No image augmentation |
| 454 | 2020 | PubMed | 10.1101/2020.12.24.424262 | DeepImmuno: Deep learning-empowered prediction and generation of immunogenic peptides for T cell immunity | No | No microscopy imaging |
| 455 | 2021 | PubMed | 10.3390/s22010167 | Early Diagnosis of Multiple Sclerosis Using Swept-Source Optical Coherence Tomography and Convolutional Neural Networks Trained with Data Augmentation | No | No microscopy imaging |
| 456 | 2021 | PubMed | 10.1364/BOE.439894 | Deep learning 2D and 3D optical sectioning microscopy using cross-modality Pix2Pix cGAN image translation | No | No image augmentation |
| 457 | 2022 | PubMed | 10.3390/jimaging8030071 | Multi-Modality Microscopy Image Style Augmentation for Nuclei Segmentation | No | No image augmentation |
| 458 | 2020 | PubMed | 10.1038/s41598-020-65716-4 | Deep learning-based hologram generation using a white light source | No | No image augmentation |
| 459 | 2020 | PubMed | 10.1007/s13534-020-00162-9 | Synthetic image augmentation with generative adversarial network for enhanced performance in protein classification | **Yes** | N/A |
| 460 | 2019 | PubMed | 10.1007/978-3-030-32239-7_23 | Active Appearance Model Induced Generative Adversarial Network for Controlled Data Augmentation | No | No image augmentation |
| 461 | 2021 | PubMed | 10.3934/mbe.2021090 | Generative adversarial network based data augmentation to improve cervical cell classification model | **Yes** | N/A |

| | | | | | | |
|---|---|---|---|---|---|---|
| 462 | 2018 | PubMed | 10.1371/journal.pone.0196846 | Optimized generation of high-resolution phantom images using cGAN: Application to quantification of Ki67 breast cancer images | No | No image augmentation |
| 463 | 2021 | PubMed | 10.12688/ f1000research.52026.2 | A deep learning segmentation strategy that minimizes the amount of manually annotated images | No | No image augmentation |
| 464 | 2020 | PubMed | 10.1155/2020/6490479 | Improved Classification of White Blood Cells with the Generative Adversarial Network and Deep Convolutional Neural Network | **Yes** | N/A |
| 465 | 2021 | PubMed | 10.1111/cmi.13280 | Improved automatic detection of herpesvirus secondary envelopment stages in electron microscopy by augmenting training data with synthetic labelled images generated by a generative adversarial network | **Yes** | N/A |
| 466 | 2022 | PubMed | 10.3390/mi13060847 | Self-Attention-Augmented Generative Adversarial Networks for Data-Driven Modeling of Nanoscale Coating Manufacturing | No | No microscopy imaging |
| 467 | 2022 | PubMed | 10.1038/s42003-022-03473-y | LSH-GAN enables in-silico generation of cells for small sample high dimensional scRNA-seq data | No | No image augmentation |
| 468 | 2022 | PubMed | 10.1093/bioinformatics/ btac652 | scSemiGAN: a single-cell semi-supervised annotation and dimensionality reduction framework based on generative adversarial network. | No | No microscopy imaging |
| 469 | 2023 | PubMed | 10.1038/s41598-023-27574-8 | Generation of highly realistic microstructural images of alloys from limited data with a style-based generative adversarial network. | No | No microscopy imaging |
| 470 | 2023 | PubMed | 10.3390/s23156930 | An Augmented Modulated Deep Learning Based Intelligent Predictive Model for Brain Tumor Detection Using GAN Ensemble | No | No microscopy imaging |
| 471 | 2023 | PubMed | 10.1088/1361-6560/acf3cb | A novel multi-frame wavelet generative adversarial network for scattering reconstruction of structured illumination microscopy | No | No image augmentation |
| 472 | 2023 | PubMed | 10.1016/j.cmpb.2023.107718 | CervixFormer: A Multi-scale swin transformer-Based cervical pap-Smear WSI classification framework | **Yes** | N/A |
| 473 | 2023 | PubMed | 10.1371/journal.pone.0283568 | Cervical cell's nucleus segmentation through an improved UNet architecture | No | No image augmentation |

| | | | | | | |
|---|---|---|---|---|---|---|
| 474 | 2023 | PubMed | 10.2196/47859 | Synthetic Tabular Data Based on Generative Adversarial Networks in Health Care: Generation and Validation Using the Divide-and-Conquer Strategy | No | No microscopy imaging |
| 475 | 2023 | PubMed | 10.1109/TMI.2023.3314695 | Digital Staining of White Blood Cells With Confidence Estimation | No | No image augmentation |
| 476 | 2023 | PubMed | 10.1016/j.media.2023.102961 | Collagen fiber centerline tracking in fibrotic tissue via deep neural networks with variational autoencoder-based synthetic training data generation | No | No microscopy imaging |
| 477 | 2023 | PubMed | 10.1021/ acs.molpharmaceut.3c00444 | Designing Cell Delivery Peptides and SARS-CoV-2-Targeting Small Interfering RNAs: A Comprehensive Bioinformatics Study with Generative Adversarial Network-Based Peptide Design and In Vitro Assays | No | No microscopy imaging |
| 478 | 2024 | PubMed | 10.1016/ j.compbiomed.2024.108046 | DSFF-GAN: A novel stain transfer network for generating immunohistochemical image of endometrial cancer | No | No image augmentation |
| 479 | 2024 | PubMed | 10.1117/1.JBO.29.3.036004 | Structurally constrained and pathology-aware convolutional transformer generative adversarial network for virtual histology staining of human coronary optical coherence tomography images | No | No image augmentation |
| 480 | 2024 | PubMed | 10.3389/fonc.2024.1300997 | Histopathology-based breast cancer prediction using deep learning methods for healthcare applications | No | No image augmentation |
| 481 | 2024 | PubMed | 10.1186/s13059-024-03338-z | scCross: a deep generative model for unifying single-cell multi-omics with seamless integration, cross-modal generation, and in silico exploration | No | No microscopy imaging |
| 482 | 2024 | PubMed | 10.1016/ j.neuroimage.2024.120674 | Gray matters: ViT-GAN framework for identifying schizophrenia biomarkers linking structural MRI and functional network connectivity | No | No microscopy imaging |
| 483 | 2024 | PubMed | 10.3171/2024.1.JNS232196 | Generation and applications of synthetic computed tomography images for neurosurgical planning | No | No microscopy imaging |
| 484 | 2024 | PubMed | 10.1016/ j.compbiomed.2024.108913 | Improving quantitative prediction of protein subcellular locations in fluorescence images through deep generative models | No | No image augmentation |
| 485 | 2024 | PubMed | 10.1021/acsami.4c11972 | On-Demand Design of Metasurfaces through Multineural Network Fusion | No | No microscopy imaging |

| | | | | | | |
|---|---|---|---|---|---|---|
| 486 | 2024 | PubMed | 10.1088/1361-6560/ad7d5b | Towards a fully automatic workflow for investigating the dynamics of lung cancer cachexia during radiotherapy using cone beam computed tomography | No | No microscopy imaging |
| 487 | 2024 | PubMed | 10.1515/bmt-2024-0028 | AML leukocyte classification method for small samples based on ACGAN | No | Not available |
| 488 | 2024 | PubMed | 10.1002/mp.17320 | A medical image classification method based on self-regularized adversarial learning | No | No microscopy imaging |
| 489 | 2024 | PubMed | 10.1080/0954898X.2024.2426580 | Kruskal Szekeres generative adversarial network augmented deep autoencoder for colorectal cancer detection | No | Not available |
| 490 | 2025 | PubMed | 10.1038/s42003-025-07469-2 | Improving 3D deep learning segmentation with biophysically motivated cell synthesis | No | No image augmentation |
| 491 | 2025 | PubMed | PMC11908362 | Self-Supervised Z-Slice Augmentation for 3D Bio-Imaging via Knowledge Distillation | No | No image augmentation |
| 492 | 2025 | PubMed | 10.1021/acs.jcim.5c00199 | CPPCGM: A Highly Efficient Sequence-Based Tool for Simultaneously Identifying and Generating Cell-Penetrating Peptides | No | No microscopy imaging |
