# Supplementary material for: Systematic review of generative adversarial networks (GANs) in cell microscopy: Trends, practices, and impact on image augmentation
Source: PLoS One. 2025 Jun 24;20(6):e0291217. doi: 10.1371/journal.pone.0291217 (PMC12186945; doi:10.1371/journal.pone.0291217)
Supplement: Attachment — Submitted filename: Response_to_Reviewers.pdf [file pone.0291217.s006.pdf]

**Rebuttal Letter - Systematic review of generative adversarial networks in cell microscopy: trends, practices, and impact on image augmentation**

We first would like to thank all the reviewers that took the time to read carefully the manuscript and gave their corrections for the sake of improving the quality of the present work. Here we will point out the changes made in the text to solve the concerns arisen during the first revision.

**1.** Is the manuscript technically sound, and do the data support the conclusions?
The manuscript must describe a technically sound piece of scientific research with data that supports the conclusions. Experiments must have been conducted rigorously, with appropriate controls, replication, and sample sizes. The conclusions must be drawn appropriately based on the data presented.

Reviewer #1: Partly
Reviewer #2: Partly

**Our Response:** We extended both the results and discussion sections to do a more in-depth analysis of the studies selected in this review. The “Beyond image generation” subsection, compiles information related to the studies which can be useful for the readers. Moreover, we used this information to discuss the repercussions of those works in future studies.

**Reviewer #1:** This article is the first systematic review of cellular GANs and summarizes and discusses the most popular methods as well as describes how to use them, which is instructive overall, but for the small number of experiments mentioned in the article that fail to reproduce the structure, this part of the experiment should probably not be ignored. And I don't think it's a very full workload for a review.

**Our Response:** Thank you very much for your insight. We decided to extend the results and discussion sections to give a more detailed insight of the studies that met the selection criteria in the review. We also included a section of good practices for GANs in image augmentation that readers can use as a starting point and minimize possible difficulties that can arise during model implementations. Furthermore, we offer these suggestions based on the results of the retrieved studies.

Unfortunately, it was not clear for us what the reviewer referred as “experiments that fail to reproduce the structure” considering that this is a systematic review and there is no experiments section. We, however, make a discussion about the experimental designs that authors normally use in their studies. This information is presented in Table 3, Table S1, and we discussed the limitations of the current practices.

**Reviewer #2:** This paper provides a review on GAN in cell microscopy for image augmentation. It is considered as a review articles which is not technical enough to suit the requirement to be published in PLOS One. Furthermore, as a review article, the analysis provided is a bit difficult to follow. Authors should provide a critical review instead of extensive descriptions. A more conclusive/novel analysis need to be done to be the main contribution of the paper.

**Our Response:** Thank you very much for your comments, we revisited our manuscript and included the following changes according to your insights:

1. Although PLOS ONE is a journal who does not accept regular review papers for publication, it does accept systematic reviews that fulfill the requirements to be considered as such. In this manuscript, we applied and followed the PRISMA 2020 statement, which are the guidelines to write systematic reviews. We would like to point out that the PRISMA statement was designed for systematics reviews in the field of health, where there are specific experimental designs that do not fit the main domain of this manuscript (deep learning). We implemented as much as possible the PRISMA 2020 statement, considering that GAN augmentation in cell microscopy has a direct impact in the health and medicine domain. Moreover, we discussed the limitations of the current statement when the main topic of the systematic review deviates from the health domain.
2. We re-arranged our manuscript to facilitate the reading. This included moving the technical definitions into the results sections and removing some information that we considered could confuse the readers.
3. We added the “Beyond image generation” subsection in the results to increase the discussion and assess the potential of GANs. We described how GANs can be implemented in different ways to achieve different goals beyond only generating synthetic samples. This addition also helped us to make a more in-depth discussion into what are the different alternatives to implementing a GAN, depending on the nature of the problem.
4. Finally, we now presented a list of good practices with the aim of helping the researchers that would like to implement GANs in the cell microscopy domain. This list seeks to increase the experiment's reproducibility and guide the research in the implementation process. Each of the points of this list come from the results and conclusions of the studies that we reviewed in this work.
