# Supplementary material for: Systematic review of generative adversarial networks (GANs) in cell microscopy: Trends, practices, and impact on image augmentation
Source: PLoS One. 2025 Jun 24;20(6):e0291217. doi: 10.1371/journal.pone.0291217 (PMC12186945; doi:10.1371/journal.pone.0291217)
Supplement: Attachment — Submitted filename: Response_to_Reviewers_auresp_2.pdf [file pone.0291217.s007.pdf]

Rebuttal Letter - Systematic review of generative adversarial networks (GANs) in cell microscopy: trends, practices, and impact on image augmentation

We first would like to thank all the reviewers that took the time to read carefully the manuscript and gave their corrections for the sake of improving the quality of the present work. Here we will point out the changes made in the text to solve the concerns arisen during the first revision.

Journal Requirements: Please review your reference list to ensure that it is complete and correct. If you have cited papers that have been retracted, please include the rationale for doing so in the manuscript text, or remove these references and replace them with relevant current references. Any changes to the reference list should be mentioned in the rebuttal letter that accompanies your revised manuscript. If you need to cite a retracted article, indicate the article's retracted status in the References list and also include a citation and full reference for the retraction notice.

Our Response: We revisited every single citation and searched it in the Retraction Watch Database to be completely sure that we were no citing retracted publications. This allowed us to update some preprints that are already published. We only found a single publication with a publisher correction that is exclusively related to the copyright license (https://doi.org/10.1038/s41592-019-0612-7), and one dataset URL link that is not available anymore. For this last citation, we decided to remove it and explain the situation in the manuscript.

1. Have the authors made all data underlying the findings in their manuscript fully available?

he PLOS Data policy requires authors to make all data underlying the findings described in their manuscript fully available without restriction, with rare exception (please refer to the Data Availability Statement in the manuscript PDF file). The data should be provided as part of the manuscript or its supporting information, or deposited to a public repository. For example, in addition to summary statistics, the data points behind means, medians and variance measures should be available. If there are restrictions on publicly sharing data—e.g. participant privacy or use of data from a third party—those must be specified.

Reviewer #1: No

Our Response: As mentioned earlier, we discovered that the URL of the Leukemia dataset is currently inaccessible. This was a dataset published in Kaggle, but for some reason is not there anymore. We removed this citation and updated the manuscript according to that. Other than that, every dataset and publication are correctly cited and can be accessed freely. Every comment and conclusion made in our manuscript is exclusively based on the publications and datasets.

Reviewer #1: The authors should describe the characteristics of the microscopic cell image data. What are the challenges in terms of processing methods compared to general image data?

Our Response: Thank you very much for your insight. We followed your comments and extended the introduction section, where we discussed the unique challenges that cell microscopy imaging has. We did not modify neither results nor discussion sections, since we already mentioned and discussed those works that directly addressed some concerns added in the introduction.
