# Supplementary material for: Systematic review of generative adversarial networks (GANs) in cell microscopy: Trends, practices, and impact on image augmentation
Source: PLoS One. 2025 Jun 24;20(6):e0291217. doi: 10.1371/journal.pone.0291217 (PMC12186945; doi:10.1371/journal.pone.0291217)
Supplement: Attachment — Submitted filename: Rebuttal_letter.pdf [file pone.0291217.s008.pdf]

Rebuttal Letter - Systematic review of generative adversarial networks (GANs) in cell microscopy: trends, practices, and impact on image augmentation

We first would like to thank the reviewer that took the time to read carefully the manuscript and gave their corrections for the sake of improving the quality of the present work. Here we will point out the changes made in the text to solve the concerns arisen during the first revision.

Reviewer #3: Generative Adversarial Networks (GANs) have received a lot of attention and have seen significant improvements since their introduction over a decade ago. However, applying GANs to specific domains, as discussed in this manuscript, present greater challenges.

Our Response: Thank you very much for all the detailed comments, this was an enormous help to improve not only the writing but also the content of our manuscript. We revised and grouped all of them, so there is no need to mention one by one.

- **Grammar and spelling:** We Corrected all the pointed grammar and spelling typos. This also includes reference inclusion within the text, and possible ambiguities from the manuscript's older version.
- **References and URL links:** We revisited and updated all the references to avoid missing fields. We also double-checked the datasets URL and confirmed that all URL links were working by the visit time. Moreover, we decided to include in both text and references the last date of access to avoid any ambiguity.
- **"As the authors are presenting a systematic review, I anticipated clearly defined research questions. However, the presentation consists of four bullet points, which are commented on throughout the manuscript. I would have expected a format comprising questions and corresponding answers (but, in fact I do not know if it is mandatory for a S.R.)":** We followed in the PRISMA statement which was designed specifically for systematic reviews. It is worth mentioning that such a protocol was designed having different fields in mind (such as psychology or medicine) and the field of computer science does not fit perfectly in all the requirements. In this case, the protocol requests the reviewers to either define research questions or objectives, and we decided to use objectives that are achieved throughout the manuscript in different ways (text, tables and good practices). This information is available in our PRISMA 2020 checklist supplementary table.
- **Preprints, their validity, and possible bias source:** There were indeed 4 publications that stayed as preprint from the 12 retrieved in both arXiv and bioRxiv. We went through the publications again and included a brief section in the text explaining that only one study published their implementation, but their architecture is an unmodified StyleGAN2-ADA, one of the most robust GANs, which achieved state-of-the-art results when it was first published. Moreover, there are more studies collected in this review implementing StyleGAN variations and achieving high scores, discarding the possibility of failure in the cell microscopy domain.

- **Vanilla GAN and CNN GAN:** We included a short paragraph explaining the definition of Vanilla GAN and how it can be used to represent loss and architectures independently to avoid possible misunderstandings. In the case of line 170-173, we did found a single study that implemented Vanilla GAN (loss and architecture), and even though it was published after 2016, the authors from that study concluded the lack of performance of GAN compared to a different baseline. On the other hand, our statements from lines 656-659 referred exclusively to the vanilla adversarial loss, so we adjust the paragraph to avoid ambiguities.

- **Citations with group of studies:** As we make a several study clustering during the results to easily explain them, we decided to follow the next criteria to make in-text citation.

  1. Studies that have information that is not easily retrieved from the Table 3.
  2. Study clusters easily retrieved from Table 3 with a size smaller than five to avoid repetitive citing
  3. Single studies that we considered to be worth mentioning (as an exemplification or by information that is unavailable in Table 3).

- **Table 2:** We updated the caption of table two to “Summary of adversarial losses”. In this case, only the Hinge adversarial loss is left to complete all the adversarial losses, but we decided not to include it since we did not find a publication that implemented it.

  We also updated the equation notation in the case of least-square loss. The min/max terms are specified only when there is ambiguity, such as on Vanilla loss where one network seeks to maximize and the other to minimize it, since a loss term is most times meant to be minimized. WGAN loss has the supremum term to the right of the equality since it is on terms of f, a term that does not directly influence W. All these equations are extracted from their original publications.

- **Discussion section:** We now linked each discussion section to its respective result section and cite the studies that exemplify the statements to facilitate the reading process. We also re-wrote ambiguous segments that lead to misunderstanding (including lines 665 and 751 from the older manuscript version).

  Re re-organized the information where we mention diffusion models, our first thought was always to show that although Diffusion is gaining attention, GANs are still competitive with different advantages over Diffusion. We expect that this section facilitates the understanding of this.

- **List of good practices:** We clarified that each point in this list can either be a conclusion from our review or from the authors of the selected studies (can be implicit conclusion). Any claim that comes from a study is correctly cited.

- **Adversarial losses section:** Here we explicitly defined Z, X, and X', and also cited accordingly the definition. We also briefly extended the definition to facilitate the understanding of this section. However, we decided not to reduce the mathematical concepts explained here because this is the core idea behind GANs, and they are the essentials to understand other GAN publications. Our idea is to give the reader the most important concepts so they can explore the field even beyond the papers we discussed in our review. We also updated the Figure 3 to facilitate the GANs definition with real examples.

  On the other side, our first draft included detailed dynamics of the data distribution during training based on the adversarial loss, but we decided to exclude them from the manuscript because then the mathematical complexity raises significantly. This is why we invited those readers interested in the math to go to Arjovsky and Bottou's work, where they make a formal and detailed dissection of the adversarial losses.

  Finally, we did not include detailed architecture figures because the core domain blocks (as in figure 3) are the most recurrent. Including layer-level architecture information and figures was out of the scope of this review.

- **Image quality metrics:** We included a short description of both the Inception Score and Fréchet Inception distance in the results section that will help the reader better understand the discussion part where we talk about the limitations of them in the field of cell microscopy.

- **Ethical implications:** We included a section within the discussion about the ethical considerations of generative models with medical imaging. We exemplified use cases, and also reference guidelines to meet ethical standards.

- **Image quality:** We followed all the formating guidelines for images and supplementary information, including PACE as the PLOS ONE tool to ensure image quality. We believe that the manuscript PDF reduces the resolution of images but a higher resolution counterpart is stores in the submission separately.
