# Supplementary material for: Systematic review of generative adversarial networks (GANs) in cell microscopy: Trends, practices, and impact on image augmentation
Source: PLoS One. 2025 Jun 24;20(6):e0291217. doi: 10.1371/journal.pone.0291217 (PMC12186945; doi:10.1371/journal.pone.0291217)
Supplement: Attachment — Submitted filename: Rebuttal_letter_auresp_4.pdf [file pone.0291217.s009.pdf]

Rebuttal Letter - Systematic review of generative adversarial networks (GANs) in cell microscopy: trends, practices, and impact on image augmentation

We first would like to thank the reviewer that took the time to read carefully the manuscript and gave their corrections for the sake of improving the quality of the present work. Here we will point out the changes made in the text to solve the concerns arisen during the first revision.

From a general perspective, we revisited the manuscript and correct possible grammar mistakes and typos from our last rebuttal, as well as updated data from the new included studies.

Reviewer #4:

1) **Since this is a systematic review, all databases containing preprints (such as arXiv and bioRxiv) should be excluded, as the research should focus solely on high-quality papers that have already undergone a peer-review process.**

Our Response: We sincerely appreciate your feedback and took it into careful consideration. While we previously defended the inclusion of preprints, we re-evaluated this decision in light of your comments and ultimately chose to retain them in our study for the following reasons:

- The PRISMA 2020 statement emphasizes transparency and completeness rather than restricting inclusion to peer-reviewed studies. Excluding preprint databases may compromise the completeness of the review, particularly in fast-moving research areas where recent developments have not yet undergone peer review.
- Our goal was not only to synthesize existing findings but also to analyze common practices in the application of GANs to microscopy. We believe that including preprints offers a more comprehensive and less biased view of current practices — both good and bad — in this multidisciplinary space where collaboration between Computer Science and Biology is essential.
- As noted in lines 167–169, only 4 out of the 15 preprints have not been peer-reviewed, and notably, the most recent two were submitted in 2024 and 2025. Excluding preprints would have resulted in the omission of up to 11 peer-reviewed studies, representing approximately 25% of all included studies. This highlights how preprint databases can help identify peer-reviewed work that might otherwise be missed due to indexing delays.

2) **Additionally, the review is limited to studies published until May 2023 (likely due to the paper revisions), but several works have been published in the past year and a half. Therefore, the trends and challenges described in the paper may have changed or should be updated.**

Our Response: We completely agree with your observation. The version you reviewed was submitted in April 2024, and while we are not aware of the reasons behind the delay in the review process, we recognize that timeliness is especially important for systematic reviews.

To address this, we updated our search to include studies published up to April 2025, identifying 14 additional eligible studies. We observed that GANs remain actively used in the field, with a steady publication rate over the past years. While not all of the new studies used public datasets, we noticed a growing trend toward their adoption, which we find encouraging.

Importantly, the main trends and patterns we identified in the 2024 version of our review still persist. The inclusion of new studies did not substantially change the statistics derived from our binary feature analysis. Therefore, our core insights remain valid in 2025, and we have updated the manuscript where relevant to reflect the new data.

3) **The authors should also provide a more thorough justification for why they chose to analyze only one type of generative model (GAN) for a specific task (augmentation), especially since such models are widely used today, particularly for domain translation (synthetic staining) or image enhancement/standardization (staining normalization).**

Our Response: Thank you for your thoughtful comment. We fully agree that transparency regarding the scope and inclusion criteria is essential, particularly in the context of the PRISMA 2020 statement. As explained in lines 100–116, we made a deliberate decision to focus solely on GAN architectures that generate samples from noise, rather than covering all possible applications of GANs in cell microscopy. We appreciate the opportunity to elaborate further:

- Tasks such as style transfer, image enhancement, or synthetic staining often fall under the category of image-to-image (I2I) translation, which, while impactful, typically require paired datasets to train effectively. In cases where unpaired I2I approaches are used, they often demand significantly larger training sets, which can be difficult to obtain in cell microscopy due to the cost and complexity of data acquisition.
- In contrast, generating images from noise does not rely on paired data and is generally more accessible to a broader range of research setups — particularly those with limited data or computational resources. This makes such models especially attractive in microscopy, where data scarcity is a common issue.
- Lastly, we chose to limit the scope in order to maintain completeness and analytical depth. Including the full range of GAN applications (e.g., I2I tasks) would have introduced a wide variety of goals and methodological considerations, making it difficult to provide a focused and coherent analysis within a single review.

We have clarified this rationale in the manuscript to better guide readers and to uphold the transparency principle of the PRISMA 2020 guidelines.
